# The severity of dissociation and dissociative phenomena in non-affective psychosis: a systematic review and meta-analyses

**DOI:** 10.1186/s12888-026-08424-z

**Published:** 2026-08-19

**Authors:** Adam O’Neill, Gillian Haddock, Kate Stalker, Kim Barnett, Amelia Pearson, Dawn Edge, Omar Laftah, Leah Millard-Brewer, Lee D. Mulligan

**Affiliations:** 1 Division of Psychology and Mental Health, School of Health Sciences, University of Manchester, Jean McFarlane Building, Manchester, M13 9PL UK; 2 Greater Manchester Mental Health NHS Foundation Trust, Manchester, UK; 3 Centre for Mental Health & Society, School of Health Sciences, Bangor University, Bangor, UK; 4 Social Care and Society, School of Health Sciences, University of Manchester, Manchester, UK; 5 Expert by Experience, Independent, UK

**Keywords:** Psychosis, Meta-analysis, Dissociation, Schizophrenia, Systematic review, Non-affective

## Abstract

**Background:**

Non-affective psychosis is associated with elevated risk of suicide, self-harm, and reduced life expectancy. Symptoms of dissociation are common in non-affective psychosis, and dissociative experiences are known to be linked to multiple markers of elevated clinical risk, including sleep disturbance and negative affect. The present review represents the first comprehensive quantification of dissociative phenomena severity across multiple instruments in non-affective psychosis.

**Methods:**

Six databases (Embase, MEDLINE, PsycINFO, PubMed, Scopus, Web of Science) were searched from inception and systematically screened. Eligibility was established against ICD/DSM criteria or service-defined categorisation, including first-episode psychosis, brief limited intermittent psychotic symptoms, and at-risk of psychosis mental states. In total, data were extracted for 22 measures. Four random effects meta-analyses were conducted for measures with three or more independent datasets: the Dissociative Experiences Scale (DES) (*k* = 51; *n* = 3,878); the Cambridge Depersonalization Scale (CDS) (*k* = 6; *n* = 351); the Examination of Anomalous Self Experience scale, using the dichotomous scoring method (EASE-D) (*k* = 11; *n* = 340); and the Examination of Anomalous Self Experience scale, using the continuous scoring method (EASE-C) (*k* = 6; *n *= 235).

**Results:**

DES data yielded a pooled mean of 21.28 (95% CI 19.19–23.38), consistent with moderately elevated dissociation. CDS data yielded a pooled mean of 36.38 (95% CI 26.89–45.87), also raised relative to community norms. EASE-D pooled means (M = 17.76 95% CI 15.29–20.24) and EASE-C pooled means (*M* = 74.86 95% CI 52.36–97.35) both indicated high and clinically relevant loads of basic self-disturbance. High heterogeneity (I^2^ > 85%) across measures highlights considerable variability in dissociative severity across samples, suggesting that levels of dissociation and dissociative experiences are not uniform in populations who experience non-affective psychosis.

**Conclusions:**

The present findings emphasise the importance of assessing dissociation in non-affective psychosis, and where necessary, intervening clinically. Nonetheless, the development of trauma-informed, dissociation-targeted clinical interventions is required to optimise approaches to support. Throughout the literature, there is a pressing need for more harmonised measurement efforts and further mechanistic research.

**Supplementary Information:**

The online version contains supplementary material available at https://doi.org/10.1186/s12888-026-08424-z.

## Introduction

Non-affective psychosis is an umbrella term which refers to a cluster of mental health conditions characterised by atypical perception, thought, and affect [1]. Contemporary diagnostic systems [2, 3] describe non-affective psychosis in terms of *positive symptoms*, such as hallucinations, delusions, and disorganised thinking; *negative symptoms*, including apathy, anhedonia, and social withdrawal; and *cognitive symptoms*, such as inattention and memory difficulties. Unlike affective psychoses, non-affective psychoses are defined by the absence of a primary mood component [4]. Schizophrenia and related non-affective diagnoses, such as schizoaffective disorder and delusional disorder, are considered prototypical non-affective psychotic disorders, and the term schizophrenia spectrum disorder (SSD), covering all those conditions, is often employed interchangeably with non-affective psychosis [5]. In clinical settings, early intervention for psychosis services and community mental health teams support individuals diagnosed with SSD, and also those experiencing first episode psychosis (FEP), brief limited intermittent psychotic symptoms (BLIPS), or who present with ultra-high-risk (UHR) indicators for developing psychosis [6]. Notably, non-affective psychosis is associated with profound psychosocial and functional impairment [7, 8], as well as markedly elevated risk of suicide, self-harm, reduced life expectancy, and excess premature mortality [9–11].

Against this background, there has been growing interest in the role of dissociative phenomena in the aetiology and phenomenology of non-affective psychosis, as well as their influence on outcomes [12, 13]. As a concept, dissociation is difficult to define [14], however, it has been found to occur across multiple psychiatric disorders [15] and appears to be characterised by certain core components, including: *derealisation*, where a person feels the world is unreal; *depersonalisation*, where a person has feelings of being outside of themselves or somehow separated from their experiences; *dissociative amnesia*, characterised by gaps in memory; *identity confusion*, where a person finds it difficult to define themselves; and *identity alteration*, where a person switches noticeably between different aspects of their personality [16–19]. In non-affective psychosis specifically, recent work has conceptualised the core subjective experience of dissociation as a “felt sense of anomaly” (FSA) [20, 21]. The investigating authors define this as “a subjective sense of strangeness” [21] which may take the form of unreality, disconnection, automaticity, or unfamiliarity, and occurs within one or more domains such as perception, identity, the body, and cognitions.

Historically, SSDs and dissociative disorders have been framed as distinct diagnostic categories [13]. Nonetheless, systematic review evidence demonstrates substantial overlap, with symptoms of dissociation frequently present in SSDs, and the positive and negative symptoms typically associated with SSDs also commonly observed in dissociative disorders [13]. In parallel, dissociative experiences appear to be elevated in schizophrenia relative to healthy controls [17], and quantitative syntheses have provided evidence that dissociation is closely linked to the occurrence of psychotic symptoms. In one meta-analysis [22], a medium sized association between dissociative experiences and positive psychotic symptoms was reported (*r* = 0.437), with significant symptom-specific relationships found for hallucinations (*r* = 0.461), delusions (*r* = 0.418), and paranoia (*r* = 0.447). These findings extend earlier work focusing on auditory verbal hallucinations, which found a large, pooled correlation between voice hearing and dissociation across clinical and non-clinical samples (*r* = 0.52) [18]. Together, this evidence supports that dissociative processes are not only common in people who experience psychosis but appear to be particularly and selectively related to positive symptoms.

Converging evidence also links dissociation in psychosis to adverse childhood experiences. A meta-analysis of this relationship in severe mental illness [19] found a medium strength association between exposure to traumatic experiences in childhood and dissociation (*r* = 0.33), with the association being strongest in SSD (*r* = 0.39). All examined forms of adversity, including physical, sexual, and emotional abuse and neglect, were positively associated with dissociation, with emotional abuse emerging as a particularly robust correlate (*r* = 0.41 in SSD). Recent psychosis-specific research replicated this finding [23], showing exactly the same level of medium association between developmental trauma and dissociation (*r* = 0.33). This pattern is consistent with broader research exploring the link between trauma and psychosis [24], with recent evidence demonstrating a dose-response relationship between cumulative childhood adversity and psychosis risk [25].

These empirical findings have stimulated theoretical models in which dissociation is positioned as a key psychological process mediating developmental trauma and psychosis. A review of mediators between childhood adversity and psychosis identified post-traumatic sequelae, including dissociation and post-traumatic stress disorder (PTSD) symptoms, as one of the main pathways through which adversity may increase psychosis risk and severity [26], as well as being candidate mechanisms underpinning the childhood trauma-psychosis association. Hence, understanding the role of dissociation in the development of psychosis may provide a potential opportunity to intervene early in the pathway to psychosis.

Despite the expanding literature on dissociation in psychosis, important gaps remain. Existing reviews have largely focused on whether dissociation and psychotic symptoms correlate, or whether dissociation mediates associations between trauma and psychosis. To date, none have systematically quantified the severity of dissociative symptoms within non-affective psychosis, nor taken an approach which is inclusive of multiple measures of dissociation and dissociative phenomena. Given parallel calls to transform psychosis services through trauma-informed and mechanism-focused interventions [27], a clearer understanding of the severity of dissociation in non-affective psychosis is clinically pressing. Exploring how pronounced dissociation is in this group, and whether dissociative experiences vary across relevant characteristics, has implications for assessment, formulation, and the development of targeted interventions, as well as for ongoing debates regarding the boundary between psychotic and dissociative symptomatology. The aim of the present research, therefore, was to establish the severity of dissociation and dissociative phenomena in non-affective psychosis and explore potential influences on severity.

## Methods

The current article reports one strand of a multi-faceted review on the nature, severity, and prevalence of dissociation in non-affective psychosis and relationships with suicide-related outcomes. A full prospective protocol for this wider review was published on the PROSPERO International Prospective Register of Systematic Reviews (CRD420251010668) [28]. This review was conducted and reported in accordance with the Preferred Reporting Items for Systematic Reviews and Meta-Analyses (PRISMA) 2020 statement [29].

Findings on suicide-related outcomes [30] and prevalence [31] are reported separately. Inclusion/exclusion criteria, search strategy, and extraction are presented for the wider systematic review [28]; however, this article reports on only the severity of dissociation and dissociative phenomena in non-affective psychosis.

Because this review did not aim to evaluate intervention effects or scope a concept qualitatively, eligibility criteria is not optimally described using established eligibility frameworks such as PICO (population, intervention, comparator, outcome) [32] or PCC (population, concept, context) [33]. In line with guidance that review frameworks should be selected according to the review question rather than applied universally [34], it was decided on a bespoke framework for this multi-faceted review. Accordingly, inclusion/exclusion criteria are presented by population, phenomena, and evidence type.

### Inclusion criteria

#### Population

(I) Individuals described as having non-affective psychotic disorders, including ICD F20–F29 ‘schizophrenia and or other primary psychotic disorders’ [3], DSM-V ‘Schizophrenia Spectrum and Other Psychotic Disorders’ [2] or classified under relevant service-defined categories, including, first-episode psychosis, brief limited intermittent psychotic symptoms, and at-risk of psychosis mental states; and (II) Clinical participants, meaning persons would meet the criteria for or be receiving treatment from mental health services (e.g., outpatient clinics, inpatient settings, early intervention services, and community mental health teams).

#### Phenomena

(I) Studies exploring dissociative phenomena in non-affective psychosis, including depersonalisation, derealisation, amnesia, identity confusion, identity alteration, detachment, absorption, and felt sense of anomaly; (II) Studies providing severity estimates of dissociation assessed using validated measures, including, but not limited to: measures of broad dissociation, e.g., the Dissociative Experiences Scale (DES) [35], measures of specific dissociative experiences, e.g., the Cambridge Depersonalization Scale (CDS) [36], measures of anomalous self-experience, e.g., the Examination of Anomalous Self Experience scale (EASE) [37], psychosis population specific measures, including the Černis Felt Sense of Anomaly Scale (ČEFSA) [21], and dissociative disorder measures, including the Structured Clinical Interview for DSM-V Dissociative Disorders, revised (SCID-D-R) [38]; (III) studies providing insight into the nature of dissociation in non-affective psychosis; (IV) studies which estimate the prevalence of dissociation in non-affective psychosis; and (V) studies which report on the role of dissociation in suicide-related outcomes in non-affective psychosis, including ideation, thoughts and behaviours, self-harm with suicidal intent, attempts, and completions.

#### Evidence type

(I) Quantitative studies, including observational studies, such as cross-sectional, cohort, and case-control studies, as well as experimental or interventionist studies that report dissociation-related measures in psychosis populations; (II) Qualitative studies that explore the experiences of dissociation in non-affective psychosis; and (III) Mixed-methods studies that combine qualitative and quantitative assessments of dissociation in non-affective psychosis.

### Exclusion criteria

#### Population

(I) Individuals described as experiencing affective psychoses (e.g., bipolar or mood disorders with psychotic experiences) or other co-morbid psychoses where non-affective psychosis or SSDs are not the primary diagnosis; and (II) Non-clinical or general population samples (e.g., student cohorts who were surveyed for psychotic-like experiences).

Non-clinical samples were excluded to ensure diagnostic coherence and finding transferability, as self-reported psychotic-like experiences are known to differ in transience, severity, and quality from the psychotic experiences observed in diagnosable psychotic disorders [39, 40]. Studies using healthy control comparators were included provided the clinical group met the review inclusion criteria. Where the sample was diagnostically mixed, these samples were included where ≥ 50% of participants aligned with non-affective psychosis diagnostic criteria.

#### Phenomena

(I) Studies which report on non-affective psychosis and/or suicide-related outcomes but do not contain a dissociation component; and (II) studies exploring non-suicidal self-harm [41].

#### Evidence type

(I) Case reports, editorials, and opinion pieces; (II) Animal studies; (III) Studies reported in a language other than English; (IV) Studies which have not been peer reviewed, including PhD theses; (V) Secondary reporting articles (e.g., systematic or narrative reviews).

### Search strategy

Six databases (Embase, MEDLINE, PsycINFO, PubMed, Scopus, Web of Science) were searched from inception to 10 December 2025. No date limits were applied, and the following search syntax was used:

(“psychosis” OR “psychotic*” OR “schizo*” OR “at-risk mental” OR “BLIPS”) AND (“dissocia*” OR “felt sense of anomaly” OR “detach*” OR “depersonalization” OR “derealization” OR “depersonalisation” OR “derealisation”) AND (“suicid*” OR “self-harm” OR “self harm” OR “suicide attempt” OR “STB” OR “prevalence” OR “epidemiology” OR “incidence” OR “rate” OR “frequency” OR “severity” OR “scale” OR “measure” OR “qualitative” OR “phenomenol*” OR “thematic”)

The reference lists of relevant systematic reviews [12, 13, 15, 17–19, 22, 26, 42–46] were also searched for pertinent citations. All retrieved references were imported into Covidence [47] software for screening, first by title and abstract, then by full text. For every article, this was conducted independently by two reviewers (AO and one of KS, AP, and KB) excepting those identified directly via reference list. A small piloting exercise was undertaken where screeners met to discuss their first ten votes and calibrate approach. After this exercise, any disagreements were resolved through discussion with study senior investigators (LM, GH) and an expert by experience collaborator (OL).

### Extraction

Data extraction was completed by AO in collaboration with other authors who monitored for accuracy at the time of quality assessment. The following data were extracted for all included articles, where available: title; country; setting (inpatient, community etc.); study design; inclusion criteria; exclusion criteria; sample size; sample characteristics; key findings; insights on the nature of dissociation in non-affective psychosis; severity score for dissociation in non-affective psychosis; measure used; prevalence estimate; and the relationship between dissociation and suicide in non-affective psychosis.

### Analysis

In line with established conventions [48, 49], meta-analysis was undertaken when at least three independent datasets (*k*) were extractable for a given validated dissociation measure. Following the two-stage inverse-variance approach [48, 49], the study mean was treated as the effect size for the study. All analyses used random effects models, reflecting an *a priori* expectation that true mean dissociation varied across samples. Analyses were conducted in R (v.4.5.1) using RStudio (v.2023.12.1). *Metafor* and *Meta* packages were used for meta-analysis and *ggplot2* was used for visualisation.

When studies reported multiple, non-overlapping subgroups drawn from the same diagnostic population, these were combined using standard fixed-effect formulas to produce a single estimate [48, 49]. For longitudinal designs, only baseline data were extracted to maintain comparability with cross-sectional studies and to minimise dependence or potential intervention effects. If studies reported 95% confidence intervals (CIs) but not standard deviations (SDs), SDs were back calculated using the standard formulas [49].

The DES [35] and DES-II [50] were treated as equivalent, as were validated translations, given their high reliability and construct equivalence [51–53]. CDS translations were also treated as equivalent, reflecting their high construct equivalence and reliability [54–56]. For EASE, separate analyses were conducted for the two adopted scoring formats. Continuous scoring (EASE-C) reflects item severity and frequency (0–5) summed into a total (0–285). Dichotomous scoring (EASE-D) classifies items as absent or present [57], yielding a total from 0-57.

Between-study variance (τ^2^) was primarily estimated using the Paule–Mandel (PM) estimator because of its favourable bias-variance performance and stability under heterogeneity [58, 59]. DerSimonian-Laird (DL) models were fitted as sensitivity analyses given their widespread implementation in one-arm meta-analyses [60]. For measures were *k* < 10, Restricted Maximum Likelihood (REML) models were additionally fit to assess estimator robustness, given evidence that REML can perform well for τ^2^ when *k* is small [61].

Hartung–Knapp (H-K) adjustments were applied to improve interval coverage where uncertainty in τ^2^ estimation was high [49, 62]. Prediction intervals (PIs) were calculated only when study numbers were sufficient to avoid substantial imprecision (*k* ≥ 10) [49, 63]. Heterogeneity was summarised using both τ^2^ and I^2^ [49]. For smaller analyses (*k* < 10), greater emphasis was placed on τ^2^ and H-K intervals when interpreting dispersion due to known instability in I^2^ at a low value *k* [61].

Small-study effects were evaluated where *k* ≥ 10 using funnel plots, Egger-type regressions [64], and a Begg-Mazumdar rank correlation [65]. Trim-and-fill analyses were also used to explore the potential impact of missing studies [66]. All bias tests were interpreted cautiously due to the susceptibility of asymmetry to reflect true heterogeneity rather than publication bias [48, 49].

Influence diagnostics involved leave-one-out analyses for all models [67]. For measures were *k* < 10, these were supplemented by Cook’s distance, DFFITS, and related influence measures, such as leverage and τ^2^ deletion, to identify studies exerting disproportionate influence on model fit [59, 67].

Subgroup analyses were conducted for measures where *k* ≥ 10 only, reflecting more adequate study numbers for comparison [68, 69]. For each subgroup, pooled mean, 95% CI, and residual heterogeneity (I^2^, τ^2^) were reported. Categorical moderators included clinical setting (inpatient, outpatient, mixed), specified diagnostic category, and country.

Meta-regression analyses were undertaken where sufficient studies were available to explore whether study-level characteristics accounted for between-study heterogeneity. Candidate moderators included clinical setting, diagnostic category, country, sample size, mean age, publication year, gender composition, Positive and Negative Syndrome Scale (PANSS) [70] total score, and Childhood Trauma Questionnaire (CTQ) [71] total score.

Moderators were first examined individually using univariable random-effects meta-regression models estimated with the PM estimator. For DES, where study numbers were sufficient to support more complex modelling, additional multivariable meta-regression models were fitted: one including country, clinical setting, and diagnostic group to examine the joint contribution of key categorical study characteristics, and a reduced combined model including clinical setting, diagnostic group, mean age, publication year, and gender composition to evaluate the independent effects of demographic and clinical moderators simultaneously whilst preserving statistical stability. For EASE-D, only exploratory univariable meta-regressions were conducted because the smaller number of studies limited the suitability of multivariable modelling.

### Quality assessment

Quality of studies was assessed using the Mixed Methods Appraisal Tool (MMAT) [72], selected as it is designed to appraise methodological quality across diverse empirical designs. The MMAT has also demonstrated good content validity and usability in systematic reviews of psychological variables [73]. Each study was assessed independently by two reviewers (AO and one of KS, AP, KB, and LM-B). A third reviewer was available to adjudicate if disagreements could not be resolved, although this process was not required for any study in the present review.

## Results

Database and citation searches initially returned 9,919 results. Following de-duplication, 4,369 unique records were screened, with 208 assessed at full text. In total, 134 studies met wider study inclusion criteria, of which 126 utilised a validated measure of dissociation.

Raw agreement between screeners was high at both title and abstract and full-text screening, with excellent inter-rater reliability demonstrated at full-text (mean Cohen’s k = 0.95) (see supplementary materials 1 and 2 for full inter-rater reliability statistics).

**Fig. 1:**
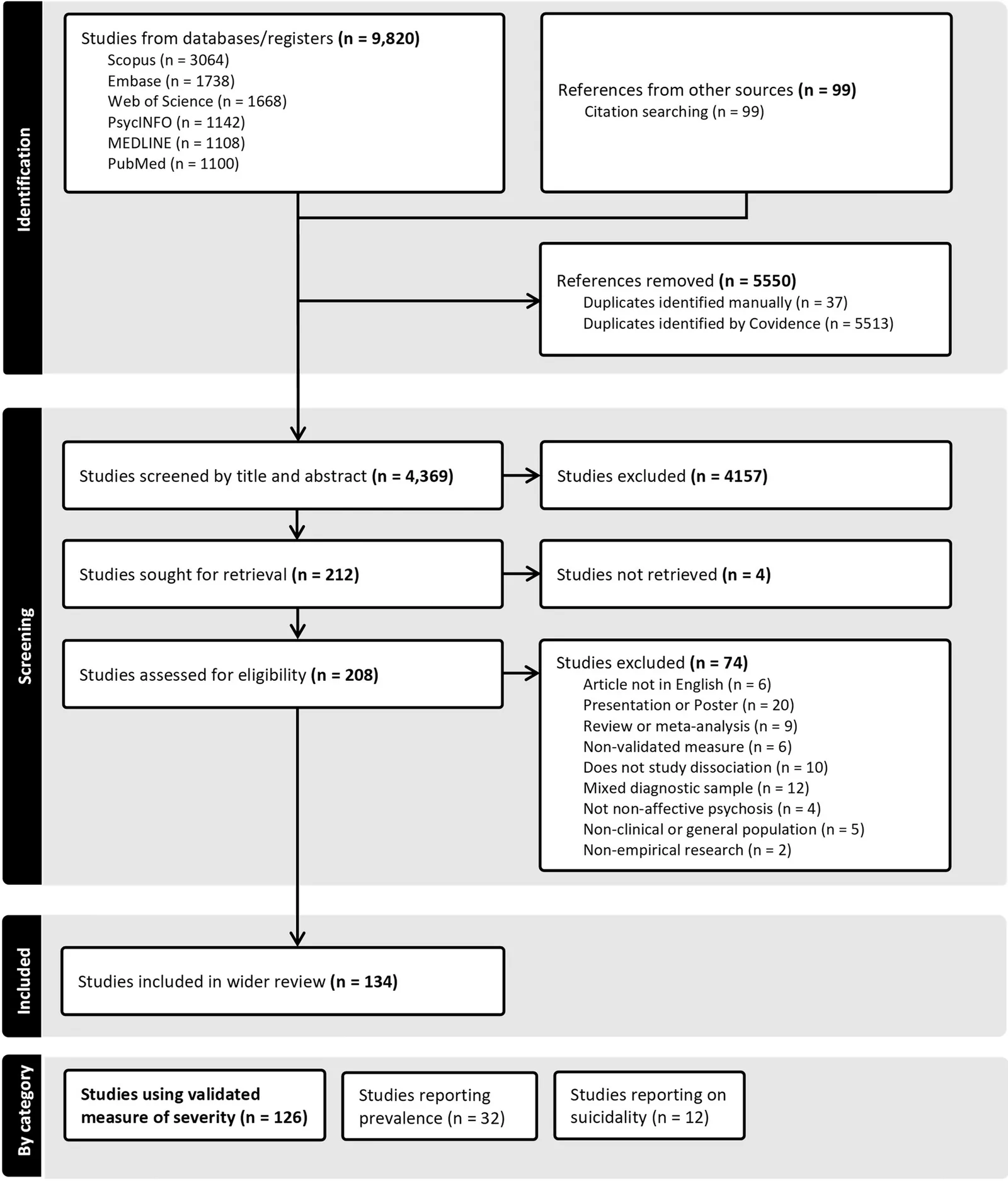
PRISMA flow diagram

A PRISMA flow diagram of study inclusion/exclusion is presented in Fig. 1.

Studies were included from 25 countries across Europe, Asia, North America, and Oceania, covering a range of characteristically diverse samples. Samples were predominantly recruited from inpatient or outpatient clinical services, and occasionally specialist settings such as early intervention or substance misuse programmes. The mean age of participants tended to cluster around the 20–45 age range, with sporadic studies falling outside of this bracket. Gender composition was almost universally reported as a male to female binary and was highly varied. Some studies recruited samples that were predominantly, or in three cases entirely, female, whereas others recruited samples that were predominantly, or in one case, entirely male. Nonetheless, studies demonstrating extreme gender skew (beyond 30–70% either way) were atypical. Most studies were either quantitative non-randomised (i.e. comparative without randomisation), or quantitative descriptive. There was one purely qualitative study, one mixed methods study, and one randomised controlled trial (RCT).

MMAT assessment found broadly clear research aims, appropriate designs, and valid measurement approaches across included studies (see supplementary materials 3 and 4 for full MMAT assessment details). However, two pervasive limitations were noted across quantitative non-randomised studies, namely, many did not utilise relevant statistical adjustment for confounders or provide adequate information to assess completeness of outcome data due to missing information on participation flow and attrition. Moreover, quantitative descriptive studies typically employed appropriate sampling approaches and analytical methods, but non-response bias was often indeterminable. Across both design types, exclusions of individuals unable to tolerate lengthy assessments, with complex co-morbidity, or in crisis, might be said to have excluded more acute non-affective psychosis presentations. However, such exclusions are in keeping with ethical standards and risk management considerations for clinically complex presentations.

Twenty-seven of the 126 studies which utilised a validated measure of dissociation employed a measure that was not meta-analysable for non-affective psychosis participants (*k* < 3). The number of measures totalled 19 and included the: AMDP Scale for Dissociation and Conversion (AMDP-DK) [74] (*k* = 2); Cognitive Appraisals of Dissociation in Psychosis scale (CAD-P) [75] (*k* = 2); Clinician-Administered Dissociative States Scale (CADSS) [76] (27-item *k* = 1; 23-item *k* = 2); Cambridge Depersonalization Scale 2-item Screener (CDS-2) [77] (*k* = 1); ČEFSA [21] (*k* = 3, overlapping samples); Depersonalization-Derealization Inventory (DDI-12) [78] (*k* = 1); Dissociative Experiences Scale, revised (DES-R) [79] (*k* = 2); Dissociation Questionnaire (DIS-Q) [80] (*k* = 2); Dissociative Symptoms Scale (DSS) [81] (*k* = 2); Dissociation Tension Scale [82] (DTS) (*k* = 1); Fragebogen zu Dissoziativen Symptomen 20 (FDS-20) [83] (*k* = 1); Global FSA Scale (G-FSA) [21] (*k* = 1); Multidimensional Inventory of Dissociation (MID) [84] (*k* = 1); Perceptual Aberration Scale (PAS) [85] (*k* = 1); Peritraumatic Dissociative Experiences Questionnaire (PDEQ) [86] (*k* = 1); SCID-D-R [38] (*k* = 1); Somatoform Dissociation Questionnaire (SDQ) [87] (*k* = 1); State Scale of Dissociation (SSD) [88] (*k* = 1); and the Tellegen Absorption Scale (TAS) [89] (*k* = 2).

These studies spanned ten countries and included 2,278 participants. A breakdown of studies, measures utilised, and a brief interpretation is provided in Table 1.

**Table 1:** Studies reporting severity score where *k* < 3

Study	Country	Setting	Diagnostic group	Sample size	Measure	Mean and SD	Interpretation
Vogel et al. [90]	Germany	Inpatients	Schizophrenia	71	AMDP Dissociation	9.6 (SD = 7.5)	Scores reported use only the AMDP-DK dissociation subscale. Whilst validation work has been undertaken in psychiatric populations [74], no threshold scores are provided. Use in non-affective psychosis appears to be limited to the works of Vogel et al., prohibiting further comparison.
Vogel et al. [91]	Germany	Community	SSD	74	AMDP Dissociation	9.81 (SD = 7.81)	
Černis et al. [75]	UK	Inpatients and Community	Non-affective psychosis	1,026	CAD-P	18.7(SD = 13.35)	Because scores originate from the same underlying sample with different missing data criteria, comparisons across independent samples are not yet possible.
Černis et al. [92]	UK	Inpatients and Community	Non-affective psychosis	902	CAD-P	18.84 (SD = 13.19	
Rajanthiran et al. [93]	Australia	Inpatients and Community	Schizophrenia	19	CADSS-23	13.83 (SD = 9.77)	Differences between the CADSS 27-item [76] and CADSS 23-item [94] versions preclude pooling of scores. Validated severity thresholds for the CADSS in non-affective psychosis are currently lacking, meaning the implications for severity and clinical meaning of CADSS values in non-affective psychosis remains uncertain.
Wearne et al. [95]	Australia	Community	Schizophrenia	44	CADSS-23	23.43 (SD = 16.86)**	
Bremner et al. [76]	US	Inpatients	Schizophrenia	22	CADSS-27	3.7 (SD = 5.3)	
Dorahy et al. [96]	New Zealand & Australia	Does not specify	SSD	45	CDS-2	0.74 (SD = 0.91)	Validation work on the CDS-2 proposed a provisional cut-off of ≥ 3 to screen for clinically significant depersonalisation in clinical samples [77]. The reported mean score reported falls well below this threshold and lies at the low end of the distribution observed in clinical groups. However, the absence of further data in non-affective psychosis samples precludes broader comparison and limits firm interpretation.
Černis et al. [75]	UK	Inpatients and Community	Non-affective psychosis	1,026	ČEFSA	39.65 (SD = 30.51)	Across several publications drawing on the same large cohort of participants with non-affective psychosis, mean scores indicate that participants reported FSA-type dissociative experiences at levels that are both elevated and highly variable. Consistently large SDs demonstrate substantial within sample variability, whilst the mean scores fall in a range that the original authors interpret as clinically relevant in the context of psychosis. Scores could not be pooled as they derive from the same underlying sample with slightly different missing-data criteria applied across reports.
Černis et al. [21]	UK	Inpatients and Community	Non-affective psychosis	1,031	ČEFSA	39.54 (SD = 30.48)	
Černis et al. [92]	UK	Inpatients and Community	Non-affective psychosis	902	ČEFSA	40.56 (SD = 30.59)	
Wearne et al. [97]	Australia	Community	Schizophrenia	10	DDI-12	1.05 (SD = 0.45)	The DDI-12 does not currently have validated clinical severity thresholds [78], and its primary psychometric work was conducted in panic disorder populations, which limits interpretive certainty for non-affective psychosis. No other DDI-12 data appear to have been published in non-affective psychosis samples.
Pearce et al. [98]	UK	Community	Schizophrenia***	82	DES-R	85.21(SD = 33.04)	The DES-R proposed screening cutoff for dissociative disorder of 71.5 [79] is exceeded by the sample of one study but not met by the sample in another. The DES-R has minimal uptake in non-affective psychosis to date, which limits opportunities for comparison and insight on these disparate scores.
Żwirska et al. [99]	Poland	Inpatients and Community	SSD	63	DES-R	41.8 (SD = 33.3)	
Akbudak et al. [100]	Turkey	Community	Schizophrenia	81	DIS-Q	2.2 (SD = 0.62)	Prior validation studies indicate that DIS-Q mean scores > 3 are typical of dissociative disorder groups, whilst healthy and mixed psychiatric samples typically average around 2–2.5 [80]. Use outside of this research group in non-affective psychosis prohibits further comparison.
Akbudak et al. [101]	Turkey	Community	Schizophrenia	42	DIS-Q	2.21 (SD = 0.53)	
Tseng et al. [102]	Taiwan	Community	Schizophrenia	37	DSS	13.7 (SD = 15.52)	Both scores are elevated compared to healthy controls (*M* = 6.3, SD = 10.1) [81]. Nonetheless, validation work in the DSS does not use a non-affective psychosis diagnostic group and no other studies were found in this population, prohibiting comparison.
Żwirska et al. [99]	Poland	Inpatients and Community	SSD	65	DSS	10.6 (SD = 10.3)	
Stiglmayr et al. [82]	Germany	Inpatients and Community	Schizophrenia	20	DTS	10.96 (SD = 5.58	The reported score is higher than healthy controls (2.79, SD = 5.58), though lower than scores observed in borderline personality (33.16, SD = 18.48) and posttraumatic stress disorder (34.41, SD = 17.98) [82]. The DTS does not appear to have been used more widely in non-affective psychosis, precluding comparison.
Tschoeke et al. [103]	Germany	Inpatients	Schizophrenia	21	FDS-20	19.9 (SD = 17.8)	The reported score falls above the validated cutoff of 13 for indication of major dissociative disorder [104]. Nonetheless, the high SD and authors’ report that only 12 of 21 participants scored above the cutoff indicate substantial within-group variability.
Černis et al. [21]	UK	Inpatients and Community	Non-affective psychosis	1,028	G-FSA	7.85 (SD = 5.61)	The G-FSA was developed using the same large cohort as the ČEFSA. No clinical cut-offs have yet been established, and uptake beyond the original development work currently remains limited, precluding wider cross-study comparison.
Laddis & Dell [105]	US	Community	Schizophrenia	40	MID	22.7 (SD = 19.9)**	No further MID data in non-affective psychosis samples were located in the wider literature, and the original authors note that this study represents the first report of MID scores in schizophrenia.
Torregrossa et al. [106]	US	Does not specify	SSD	152	PAS	6.43 (SD = 6.27)	The reported mean is similar to that reported in the original validation work in schizophrenia (*M* = 5.71, SD = 5.89, *n* = 66) [85] and scores during initial psychiatric admission under various diagnoses (range: 5.79–7.67, SD = 5.72–7.56) [107]. At present, there is no empirically validated cut-off or severity threshold for PAS scores.
Ma et al. [108]	China	Inpatients	Schizophrenia	71	PDEQ	14.84 (SD = 6.4)**	This score is significantly heightened compared to general population samples exposed to trauma, including help-seeking members of the public (*M* = 3, SD = 1) and non-help-seeking members (*M* = 2.2, SD = 1) [109]. However, more widely, the PDEQ is typically employed in post-traumatic stress disorder groups or general populations exposed to trauma [110, 111], meaning its use in non-affective psychosis is minimal.
O’Connell et al. [112]	Australia	Does not specify	Psychotic disorder	58	SCID-D-R	9.67 (SD = 3.67)	Whilst higher scores indicate greater and recurrent levels of psychopathology [38], no cut-offs are currently given to stratify SCID-D-R scores, and limited uptake reporting in non-affective psychosis samples prohibits extensive comparison. O’Connell et al. report that 32.76% of participants meet criteria for a dissociative disorder, but do not specify a numeric cut-off or explicit diagnostic rationale for this estimation.
Sun et al. [113]	Australia	Community	Psychotic disorder	66	SCID-D-R	7.24 (SD = 2.7)	
Dogan et al. [114]	Turkey	Does not specify	Schizophrenia	48	SDQ	29.5 (SD not extractable)**	The reported score falls above scores observed in bipolar and mood disorder (*M* = 21.6, SD = 1.93), eating disorders (*M* = 27.7, SD = 8.77), and is very close to scores in somatoform disorders (*M* = 31.9, SD = 9.37) [87], suggesting highly clinically relevant somatoform dissociative experiences are present in non-affective psychosis. Nonetheless, limited use of the SDQ in non-affective psychosis precludes wider comparison.
Żwirska et al. [99]	Poland	Inpatients and Community	SSD	64	SDQ	27.2 (SD = 8.4)	
Kruger & Mace [88]	UK	Inpatients and Community	Schizophrenia	18	SSD	2.10 (SD = 1.69****)	Validation analyses of the SSD identified a provisional threshold of ≥3.9 for increased probability of dissociative disorder [88]. The reported mean score falls below this threshold. The absence of further data in non-affective psychosis samples precludes broader comparison and limits firm interpretation.
Escudero-Perez et al. [115]	Spain	Inpatients	Schizophrenia***	55	TAS (continuous)	48.89 (SD = 26.67)	Escudero-Perez et al. and Perona-Garcelan et al. adopted a Likert-scale continuous scoring method whilst Rosen et al. adopted a dichotomous scoring approach. Population norms are not currently available for the TAS and validation work in non-affective psychosis populations does not report unadjusted TAS means [116], therefore, comparison with other studies is not currently possible.
Perona-Garcelan et al. [117]	Spain	Community	SSD	62	TAS (continuous)	47.98 (SD = 25.7)	
Rosen et al. [118]	US	Community	Schizophrenia	44	TAS (dichotomous)	16.11 (SD = 8.50)	

**pooled score from subgroups; ***majority diagnosis; ****derived from confidence intervals (CIs)

Across these studies, dissociation in non-affective psychosis was assessed using a wide range of instruments that were often developed for use within other diagnostic groups and, in many cases, had not been validated for use in psychosis. This measurement variation made it difficult to compare findings across papers, interpret what “high” or “low” scores mean clinically, or merge results in any meaningful way. These restrictions were further amplified by small study numbers and the absence of psychosis-specific severity thresholds across measures. Efforts to develop tools for use in psychosis experiencing populations are thus an important advancement. Work on felt sense of felt sense of anomaly type dissociation (ČEFSA and related screeners) offers a more targeted approach and has found consistently elevated, highly variable scores within a large clinical cohort. However, because this evidence comes from a single, albeit very large, underlying sample, further replication and wider uptake are needed to establish robustness and to clarify how score levels should be interpreted beyond this sample.

Despite this fragmented picture, multiple measures were in fact sufficiently utilised within non-affective psychosis to be meta-analysable. These measures included the DES (*k* = 51), CDS (*k* = 6), and EASE, analysed using both the dichotomous (EASE-D; *k* = 11) and continuous scoring methods (EASE-C; *k* = 6).

Before analyses were performed, a number of studies included in the wider review were removed from quantitative pooling as, despite employing a validated measure of dissociation, data required for the current meta-analysis could not be extracted (*k* = 21). Reasons included: missing variability statistics (*k* = 5); no total mean reported (*k* = 8); value outside of scale ranges (*k* = 2); only adjusted means reported (*k* = 2); selective sample for high dissociation (*k* = 1); measure modified (*k* = 1); or non-separable diagnostic subsamples (*k* = 2). Additional exclusions were made to avoid double-counting participants where papers reported scores from overlapping samples (*k* = 7). Of these, Haug et al. [119] was chosen for inclusion as this paper reported scores for a full baseline sample, whereas four subsequent papers [120–123] reported scores for smaller, more specific subsets. Two publications [124, 125] reported data from FEP samples recruited in the same time period, from overlapping clinical settings in the same geographical region. Because participant overlap could not be ruled out, only the publication with the larger sample was included [124]. Giesbrecht et al. [126] and Merckelbach et al. [127] reported the same scores for the same schizophrenia sample; Giesbrecht et al. [126] was selected as the more recent publication. Likewise, Alvarez et al. [128] and Alvarez et al. [129] also reported on the same sample, with the more recent publication [129] retained. Finally, Grady et al. [130] was retained over a companion article [131] drawing on the same data set and reporting the same DES scores, as Grady et al. [130] focused on dissociation’s role in psychosis more broadly, whereas the companion article adopted a focus around influences on negative symptoms.

### Meta-analysis of the severity of dissociation in non-affective psychosis by DES score

A total of 51 studies across 18 countries were included in the meta-analysis, comprising 3,878 participants with non-affective psychosis. Across reporting studies, the sample-size weighted mean age was 36.6 years. The studies were published over a broad time span, ranging from 1988 to 2025. Gender distribution was reported in most studies, with the weighted proportion of male participants being 56.8%. Samples were drawn from both inpatient, community, and mixed inpatient-community settings. Details for the studies included in the DES meta-analysis are presented in Table 2.

**Table 2:** Studies included in DES meta-analysis

Study	Country	Clinical setting	Diagnostic grouping	Sample size	DES mean and SD	Mean age	PANSS mean	CTQ mean	%Male
Akbey et al. [132]	Turkey	Community	Schizophrenia	100	22.62 (SD = 17.62)	38.7		45.17	69
Alvarez et al. [129]	Spain	Community	SSD	45	18.6 (SD = 16.3)	41.4			55.6
Bob et al. [133]	Czechia	Community	Paranoid Schizophrenia	58	14.21 (SD = 11.17)	27.8			51.7
Bozkurt Zincir et al. [134]	Turkey	Inpatients	SSD	54	23.35 (SD = 23.7)	35.07	62.51	44.16	0
Braehler et al. [135]	Canada	Inpatients	FEP and Psychotic disorder	105	16.51 (SD = 16.33)**				
Castillo et al. [54]	Spain	Inpatients and Community	Schizophrenia	77	21.73 (SD = 20)	35			64.9
Chae et al. [136]	South Korea	Inpatients	Schizophrenia	98	18.9 (SD = 17.5)	43	65.7	48.9	51
Degnan et al. [137]	UK	Inpatients and Community	Schizoaffective***	242	37.41 (SD = 24.07)	33.17			63.6
Ghaffarinejad et al. [138]	Iran	Does not specify	Schizophrenia	50	20.43 (SD = 1.3)	32.24			72
Giesbrecht et al. [126]	Netherlands	Inpatients	Schizophrenia	22	21.52 (SD = 16.49)	38.15			
Goff et al. [139]	US	Community	Psychotic disorder	62	15.8 (SD = 14.5)**	42			65.6
Grady et al. [130]	Ireland	Community	SSD***	71	17.68 (SD = 14.27)	43			59.2
Jeong et al. [140]	South Korea	Inpatients and Community	Schizophrenia	552	22.24 (SD = 17.7)**	44.9			54.7
Justo et al. [141]	Spain	Inpatients, Community and Forensic	Schizophrenia	120	16.96 (SD = 15.47)**				77.2
Laferriere-Simard et al. [142]	Canada	Inpatients and Community	SSD	50	18.12 (SD = 12.68)	32			44
Lipschitz et al. [143]	US	Community	Psychotic disorder	18	17.88 (SD = 16.34)				26
Longden et al. [144]	UK	Community	FEP	67	23.78 (SD = 17.99)**	25.31			50.7
Longden et al. [145]	UK	Community	SSD	40	29.5 (SD = 20.4)**				
Luque-Luque et al. [146]	Spain	Inpatients and Community	Paranoid Schizophrenia	48	22.01 (SD = 16.48)	33.7	75.29		68.8
Messina [147]	Italy	Community	Schizophrenia	32	24.8 (SD = 11.2)	35.2			65.6
Modestin et al. [148]	Switzerland	Community	SSD	43	9.9 (SD = 6.8)	30			58
Mohammadzadeh et al. [149]	Iran	Does not specify	Schizophrenia	70	37.48 (SD = 13.09)**	32.77			57.1
Nesbit et al. [150]	New Zealand & Australia	Inpatients and Community	SSD	49	24.33 (SD = 21.08)	45.14			57.1
Offen et al. [151]	UK	Community	Psychotic disorder	26	25.95 (SD = 15.23)	34			
Oh et al. [152]	South Korea	Inpatients and Community	Schizophrenia	68	17 (SD = 18.1****)	34.5			38.2
Panov [153]	Bulgaria	Inpatients	Schizophrenia	105	29.36 (SD = 22.39)	36.98	62.97		
Pec et al. [154]	Czechia	Community	Schizophrenia	31	13.7 (SD = 10.79)*	36.2			48.4
Pec et al. [155]	Czechia	Community	Schizophrenia	36	14 (SD = 10.59)*	37.7			50
Pec et al. [156]	Czechia	Community	Schizophrenia	40	16.04 (SD = 11.47)*	38.6			47.5
Perona-Garcelan et al. [157]	Spain	Community	SSD	51	16.91 (12.46)**	37.18			80.4
Perona-Garcelan et al. [158]	Spain	Community	Paranoid Schizophrenia***	71	18.7 (SD = 13.42)	39.08			68.5
Plazzi et al. [159]	Italy	Inpatients	Schizophrenia	21	16.33 (SD = 12.83)	36.81	41.82		71.4
Putnam et al. [160]	US & Canada	Inpatients and Community	Schizophrenia	55	17.6 (SD = 16)	31.6			
Ricci et al. [124]	Italy	Inpatients and Community	FEP	70	29.5 (SD = 10.4)**	22.55	78.8		50
Ricci et al. [161]	Italy	Inpatients	SSD	61	31.1 (SD = 8.7)**	23.98			49
Ricci et al. [162]	Italy	Inpatients	SSD	108	32 (SD = 9.64)**	24.76			49.07
Ross et al. [163]	Canada	Community	Schizophrenia	20	17.1 (SD = 15.3)				
Sahu et al. [164]	India	Inpatients and Community	Schizophrenia	60	14.67 (SD = 9.9)		54.2		50
Sar et al. [165]	Turkey	Inpatients and Community	SSD	70	18.1 (SD = 16.6)	38.3		43.6	45.7
Schafer et al. [166]	Germany	Inpatients	Schizophrenia	15	21.0 (SD = 17.7)	34.6	75.6	48.5	0
Schafer et al. [167]	Germany	Inpatients	SSD	83	19.2 (SD = 15)	34	80.3		67
Schlesselmann et al. [168]	Netherlands	Does not specify	SSD	248	13.87 (SD = 13.23*)	40.77			62
Schroeder et al. [169]	Germany	Inpatients	SSD	145	14.1 (SD = 12)	34	66.1		67
Stone et al. [170]	US	Community	SSD	47	17.9 (SD = 16.6)	33.6			63.8
Sun et al. [113]	Australia	Community	Psychotic disorder	66	25 (SD = 16.41)	20.18	49.64	46.77	42.4
Tseng et al. [102]	Taiwan	Community	Schizophrenia	37	18.13 (SD = 17.39)	35.7	55.76		56.8
Uyan et al. [171]	Turkey	Community	Schizophrenia	100	21.47 (SD = 13.3)	42.38		53.92	49
Varese et al. [24]	UK	Inpatients and Community	SSD	45	30.25 (SD = 14.4)**				53.3
Varese et al. [172]	UK	Community	SSD	19	54.12 (SD = 13.93)	28.1			42.1
Vogel et al. [173]	Germany	Inpatients	Schizophrenia	87	15.3 (SD = 14.1)	39			64.4
Yargic et al. [174]	Turkey	Inpatients and Community	SSD	20	15.6 (SD = 2.7)	31.7			45

*score provided by authors; **pooled score from subgroups; ***majority diagnosis; ****derived from confidence intervals (CIs)

**Fig. 2:**
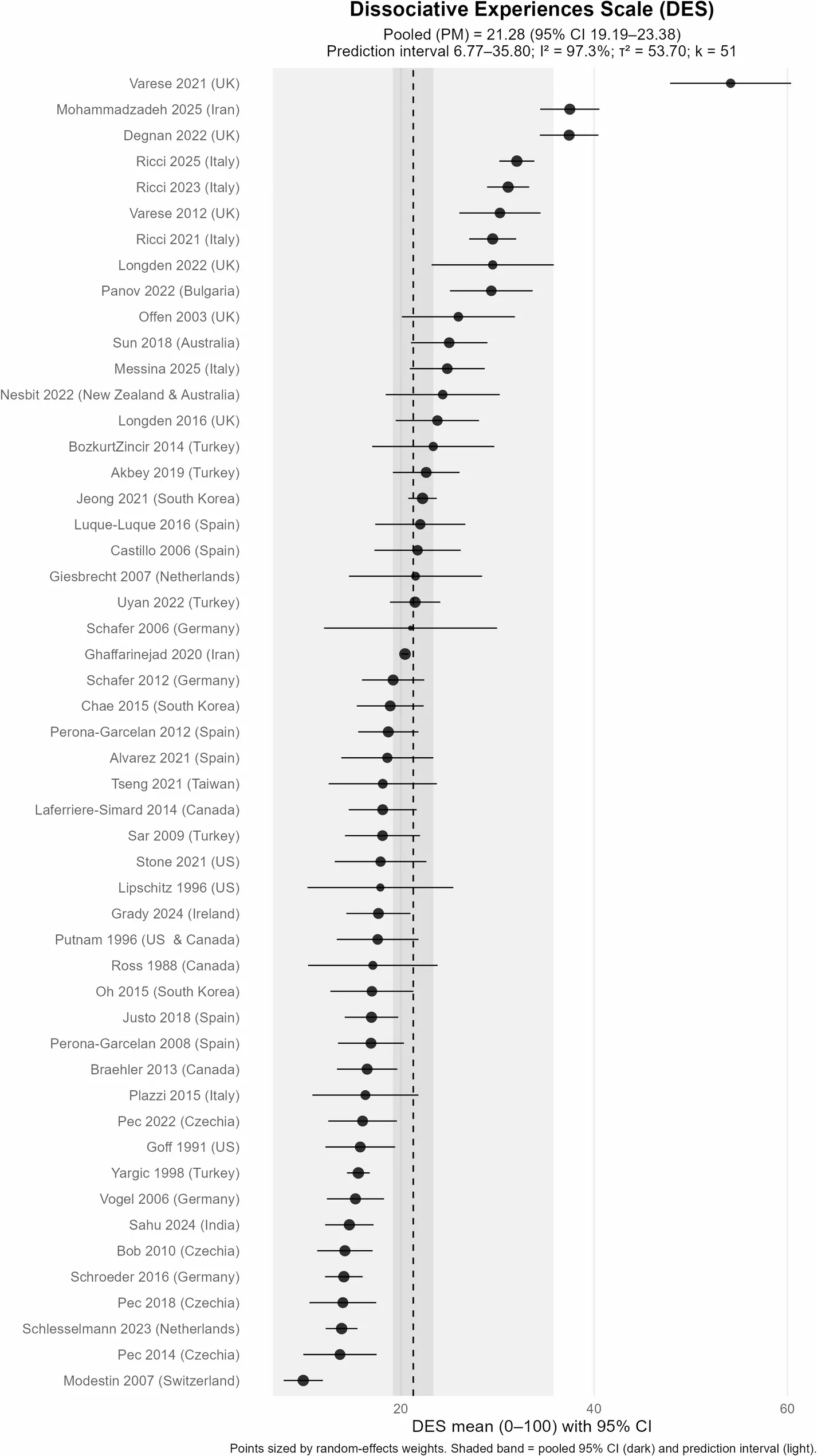
DES forest plot

Using a random-effects model with the PM estimator, the pooled mean DES score was 21.28 (95% CI 19.19–23.38), indicating that, on average, individuals with non-affective psychosis report moderate dissociative symptoms. Notably, between-study heterogeneity was substantial (I² = 97.29%, τ² = 53.70), reflecting wide variation in symptom severity. The corresponding 95% PI ranged from 6.77 to 35.80, suggesting that the mean DES score in a new, comparable study could plausibly fall anywhere within this broad range. A forest plot for meta-analysed DES studies is presented in Fig. 2.

The sensitivity analysis using the DL estimator yielded a highly similar pooled mean of 21.24 (95% CI 19.59–22.89; I^2^= 95.51%), confirming that the results were not dependent on the choice of τ^2^ estimator. Leave-one-out influence analysis further demonstrated the stability of this estimate. When each study was sequentially omitted, the pooled estimate varied minimally between 20.68 and 21.52, indicating that removal of any single study produced only modest changes in the overall estimate. This stability strengthens confidence in the central pooled value and suggests that the high heterogeneity observed is not attributable solely to any one study.

**Fig. 3:**
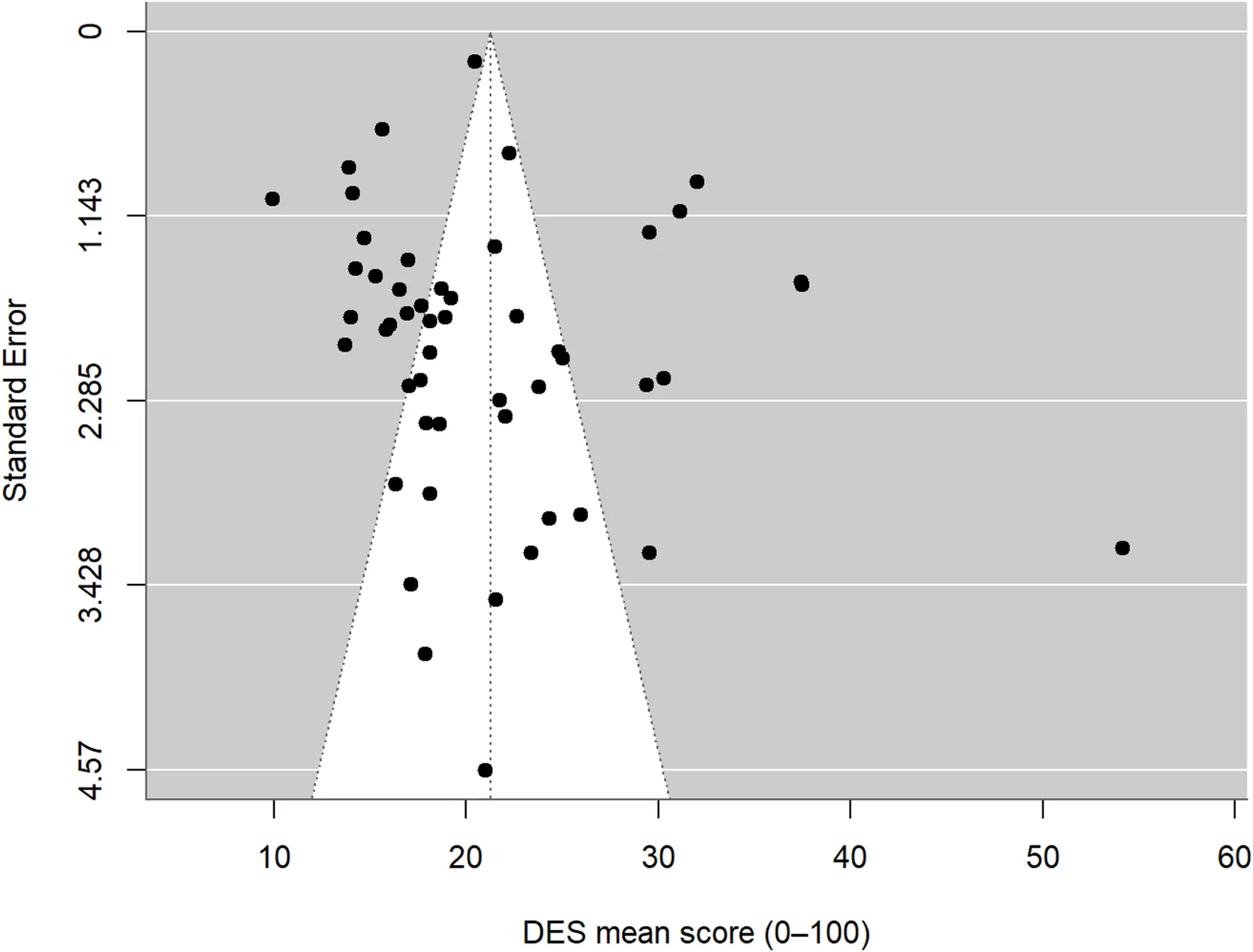
DES funnel plot

Visual inspection of the funnel plot indicated some right-sided asymmetry, driven largely by a small number of studies reporting comparatively high mean DES scores, including one particularly highly scoring study. An Egger-type regression test was non-significant (z = 1.25, *p* = 0.211), whilst a Begg–Mazumdar rank correlation was weakly positive and approached conventional statistical significance (τ = 0.19, *p* = 0.053), suggesting at most tentative evidence of a small-study effect. Trim-and-fill analysis did not impute any missing studies (k₀ = 0). The adjusted pooled mean remained unchanged at 21.28 (95% CI 19.19–23.38), indicating that correction for potential publication bias did not materially affect the overall estimate. Although statistical power for detecting publication bias is limited in single group mean meta-analyses, the combined graphical and quantitative evidence does not provide clear evidence that publication bias materially affected the observed findings. A funnel plot for meta-analysed DES studies is presented in Fig. 3.

Subgroup analyses indicated that by clinical setting, pooled mean DES scores were broadly similar across community (20.46, 95% CI 16.84–24.07; I^2^ = 95.02%; *k* = 22) and inpatient (21.61, 95% CI 17.96–25.25; I^2^ = 93.47%; *k* = 12) samples. Studies spanning both inpatient and community contexts showed comparable means (22.18, 95% CI 18.51–25.85; I^2^ = 95.69%; *k* = 13). Studies that did not specify inpatient or community setting yielded a higher pooled mean (23.87, 95% CI 10.11–37.63; I^2^ = 99.61%; *k* = 3), but the very small number of studies and wide CI substantially limit the interpretability of this estimate. Overall, these results suggest that clinical setting is unlikely to drive between-study differences in dissociative symptom severity.

By assigned diagnostic grouping, dissociation appeared highest in samples grouped under the term FEP (26.93, 95% CI 21.35–32.50; *k* = 2), compared with samples defined by the label schizophrenia (19.95, 95% CI 17.61–22.29; *k* = 22). SSD defined samples showed intermediate pooled means (22.59, 95% CI 17.68–27.50; *k* = 17), as did psychotic disorder defined samples (21.17, 95% CI 16.12–26.23; *k* = 4), whereas samples defined by the diagnosis paranoid schizophrenia yielded lower pooled means (18.03, 95% CI 13.65–22.41; *k* = 3). Whilst the small number of studies utilising some diagnostic labels seriously limits the interpretability of these comparisons, more fundamentally, these comparisons should be interpreted with particular caution given the substantial overlap between diagnostic categories in non-affective psychosis. Persistently high heterogeneity across all diagnostic categories (I^2^ = 76–98%) further indicates that assigned diagnostic classification alone fails to meaningfully organise variability in dissociative symptom severity.

By country, pooled mean DES scores ranged from 14.47 in Czechia (*k* = 4) to 33.44 in the UK (*k* = 6). Intermediate pooled means were observed in Spain (18.65, *k* = 6), Turkey (19.74, *k* = 5), and the US (16.74, *k* = 3), with higher pooled means reported from Italy (27.11, *k* = 5). No single country contributed more than six studies, several contributed only two or three, and those represented by a single study were excluded from subgroup analysis. As a result, statistical precision for these estimates is limited, and they should be interpreted cautiously.

Exploratory univariable meta-regressions showed that most continuous study-level characteristics explained little heterogeneity. Mean age and publication year were modest but statistically significant predictors. Specifically, mean age was negatively associated with DES scores (β = −0.479, 95% CI −0.852 to −0.106, *p* = 0.012; R^2^ = 12.03%; *k* = 44), and publication year was positively associated with DES scores (β = 0.313, 95% CI 0.092 to 0.535, *p* = 0.005; R^2^ = 12.97%; *k* = 51), indicating that studies with younger participants and those published more recently tended to report slightly higher dissociation levels. In contrast, sample size (β = 0.005, 95% CI −0.021 to 0.030, *p* = 0.762; R^2^ = 0%; *k* = 51), PANSS total (β = 0.136, 95% CI −0.125 to 0.397, *p* = 0.306; R^2^ = 0.50%; *k* = 12), CTQ total (β = 0.004, 95% CI −0.539 to 0.547, *p* = 0.989; R^2^ = 0%; k = 7), and gender composition (β = −0.069, 95% CI −0.222 to 0.084, *p* = 0.375; R^2^ = 0%; *k* = 45) were non-significant. Effect values for PANSS and CTQ scores should be interpreted especially cautiously given the small number of studies reporting these values.

To examine whether study characteristics jointly accounted for heterogeneity, a multivariable meta-regression including country, setting, and assigned diagnostic group was subsequently fitted. This model explained 32.28% of between-study heterogeneity, with substantial residual heterogeneity remaining (τ^2^ = 36.37, I^2^ = 92.47%). The omnibus test of moderators was statistically significant (QM(28) = 48.93, *p* = 0.009), indicating that these variables collectively contributed to explaining some of the variation in mean DES scores. Nonetheless, no single moderator showed a consistent independent effect, and substantial residual heterogeneity persisted, indicating that much of the variability likely arises from unmeasured or interacting study-level factors.

A final, reduced combined model incorporating setting, assigned diagnostic group, mean age, publication year, and gender composition (*k* = 40) explained 19.25% of heterogeneity and the omnibus test of moderators approached, but did not reach, statistical significance (QM(12) = 20.43, *p* = 0.059). Substantial residual heterogeneity persisted (τ^2^ = 49.34, I^2^ = 95.08%), with publication year being the only significant independent predictor (β = 0.477, 95% CI 0.114–0.839, *p* = 0.010). Although both age and publication year were significant in univariable analyses, only publication year remained significant following adjustment for other study characteristics. Overall, the examined moderators accounted for only a modest proportion of between-study heterogeneity, and the persistence of substantial residual heterogeneity suggests that additional unmeasured factors are likely contributing to variability in dissociation severity across studies.

### Meta-analysis of the severity of depersonalisation and derealisation in non-affective psychosis by CDS score

A total of six studies were included in the meta-analysis of depersonalisation/derealisation (DP/DR) severity measured using the CDS, comprising 351 participants with non-affective psychosis. Studies were conducted across two countries (five in Spain, and one in the UK) and were published between 2011 and 2020. Samples were drawn from a range of clinical contexts, including inpatient, community, and mixed inpatient-community settings. Details for the studies included in the CDS meta-analysis are presented in Table 3.

**Table 3:** Studies included in CDS meta-analysis

Study	Country	Setting	Diagnostic group	Sample size	CDS Mean and SD
Castillo et al. [54]	Spain	Inpatients and community	Schizophrenia	77	43.16 (SD = 37)
Černis et al. [175]	UK	Does not specify	Non-affective psychosis	50	36.8 (SD = 22)
Escudero-Perez et al. [115]	Spain	Inpatients	Schizophrenia	55	22.4 (SD = 19.53)
Luque-Luque et al. [146]	Spain	Inpatients and community	Paranoid Schizophrenia	48	40.58 (SD = 32.7)
Perona-Garcelan et al. [176]	Spain	Community	Paranoid Schizophrenia	59	53.92 (SD = 38.95)
Perona-Garcelan et al. [117]	Spain	Community	SSD	62	24.47 (SD = 22.2)

**Fig. 4:**
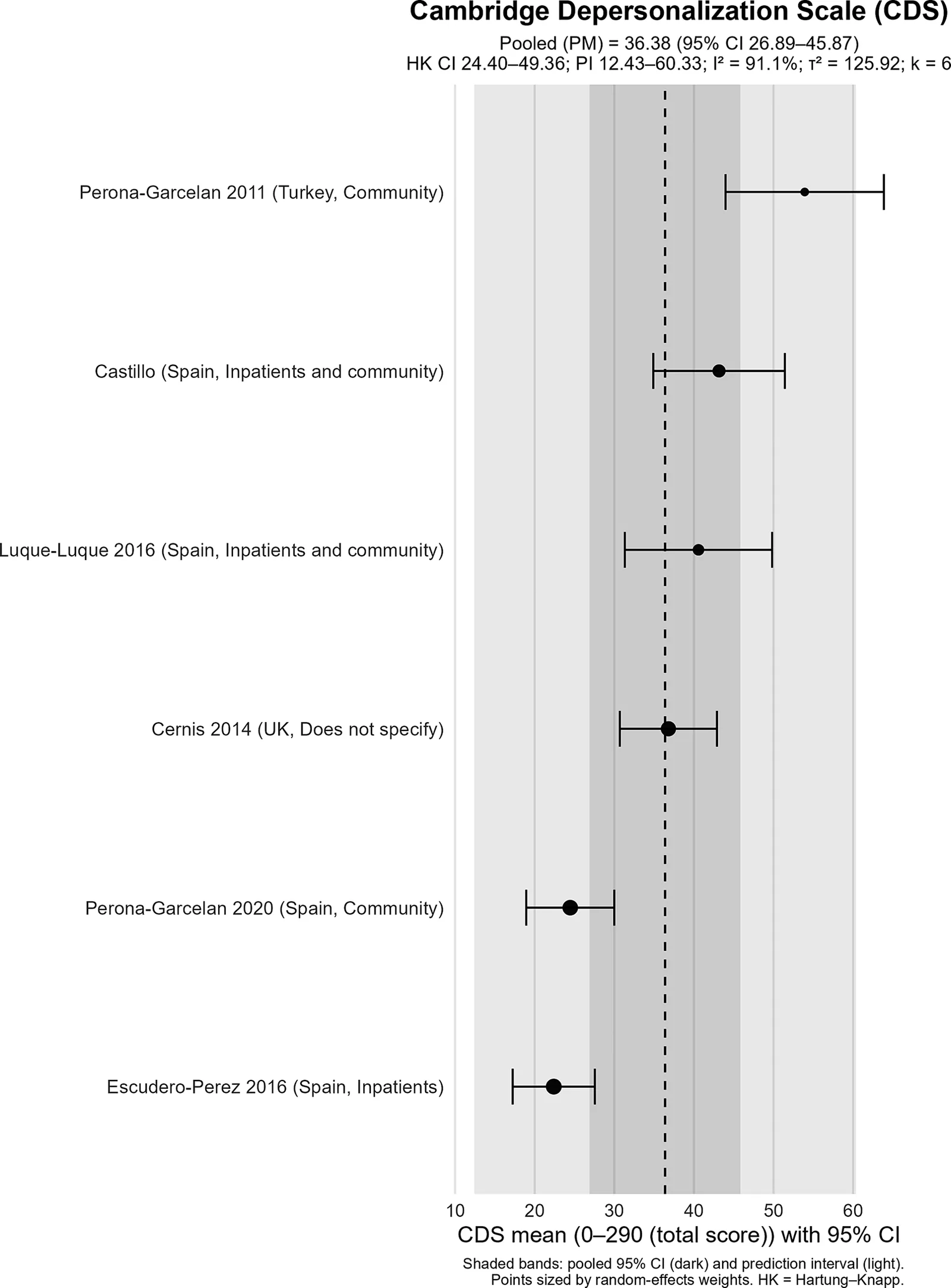
CDS forest plot

A random-effects model using the PM estimator yielded a pooled mean CDS score of 36.38 (95% CI 26.89–45.87). This is above typical community levels but below the ≥70 threshold widely used to indicate clinically significant DP/DR, and even further below means observed in DP/DR disorder (DDD) [36]. Between-study heterogeneity was substantial (τ^2^ = 125.92; I^2^ = 91.13%), indicating that the magnitude of DP/DR varied across samples. A H-K adjustment provided a similar yet slightly wider CI (23.59–51.98). A forest plot for meta-analysed CDS studies is presented in Fig. 4.

Sensitivity analyses demonstrated excellent stability of the pooled estimate across variance estimators (PM: 36.38, 95% CI 26.89–45.87; REML: 36.38, 95% CI 26.93–45.83; DL: 36.47, 95% CI 27.05–45.89), indicating that the findings were not dependent on the choice of τ^2^ estimator. Leave-one-out diagnostics showed pooled mean CDS scores ranging from 34.4 to 38.9, with confidence intervals overlapping in every iteration, and the overall mean shifting by less than ±2 points across removals.

Visual inspection of influence diagnostics indicated that Perona-Garcelan et al. [176] exerted the greatest leverage relative to other studies, consistent with a comparatively higher mean score. However, exclusion of this study reduced the pooled mean only marginally from 36.38 to 34.4, and no study exceeded conventional thresholds for influential outliers. Collectively, the robustness tests confirm that the pooled estimate is unlikely to be driven by any single study and that the substantial heterogeneity likely reflects genuine between-sample variability.

### Meta-analysis of the severity of anomalous self-experience in non-affective psychosis by EASE-D score

A total of ten studies with 11 independent data sets were included in the meta-analysis of anomalous self-experience severity, measured using the dichotomous scoring method. Together, they comprised 340 participants with non-affective psychosis and service-defined clinical risk states. Studies were conducted across six countries (Norway, France, Italy, Australia, Denmark, and South Korea) and were published between 2012 and 2025. Across reporting studies, the sample-size weighted mean age was 24.2 years, and a weighted gender distribution proportion of 55% male participants. Samples were drawn from inpatient, community, and mixed inpatient/community settings, reflecting a range of clinical contexts. Details for the studies included in the EASE-D meta-analysis are presented in Table 4.

**Table 4:** Studies included in EASE-D meta-analysis

Study	Country	Setting	Diagnostic group	Sample size	EASE Dichotomous Mean and SD	Mean age	PANSS Mean	%Male
Haug et al. [119]	Norway	Inpatient and community	Schizophrenia	57	25.3 (SD = 9.6)	25.4	76.4	51
Martin et al. [177]	France	Community	Schizophrenia	28	15.4 (SD = 7.7)	31	74.1	82.1
Mosca et al. [178]	Italy	Inpatient and community	SSD	30	15.67 (SD = 6.85)	27.87	57.23	90
Nelson et al. [179]	Australia	Community	FEP	16	16.38 (SD = 11.94) **	21.63		62.5
Nordgaard & Parnas [180]	Denmark	Inpatient	Non-affective psychosis	46	19.63 (SD = 8.39)	26.5		37
Nordgaard et al. [181]	Denmark	Inpatient	Non-affective psychosis***	48	20.63 (SD = 8.14)	25.5		37.5
Park et al. [182]	South Korea	Community	UHR	22	12.5 (SD = 9.2)	21.8		73
Raballo & Parnas [183]	Denmark	Inpatient	Schizophrenia	19	21.4 (SD = 9.6)	25.7	55.9	36.8
Raballo et al. [184]	Italy	Does not specify	Early onset schizophrenia-spectrum psychosis / UHR	13 / 23	21 (SD = 6.6) / 12 (SD = 4.5)	15.7 / 15.6		38.5 / 47.8
Vaernes et al. [185]	Norway	Community	UHR	38	15.45 (SD = 8.31)	19.8		63

**pooled score from subgroups; ***majority diagnosis

**Fig. 5:**
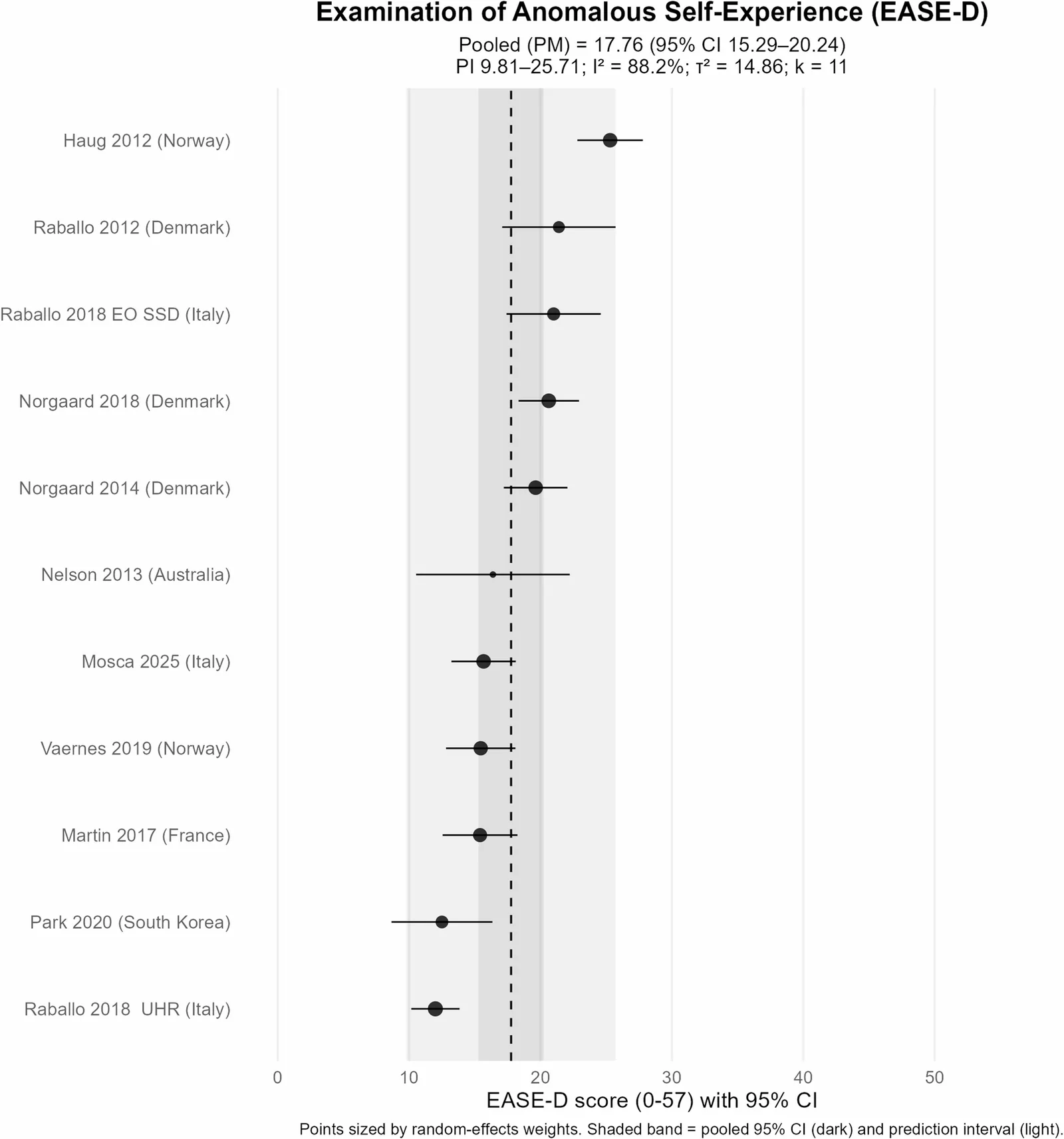
EASE-D forest plot

Using a random-effects model with the PM estimator, the pooled mean EASE-D score was 17.76 (95% CI 15.29–20.24), indicating a substantial, clinically relevant load of self-disturbance experiences in non-affective psychosis. This is a high score exceeding those reported in other mental health conditions and healthy controls [186]. Moreover, between-study heterogeneity was substantial (I^2^ = 88.15%, τ^2^ = 14.86), underscoring considerable variability across data sets. The corresponding 95% PI was 9.81 to 25.71, suggesting that the mean EASE-D score in a comparable future study could plausibly fall anywhere within this broad range. A forest plot for meta-analysed EASE-D studies is presented in Fig. 5.

Sensitivity analyses using the DL estimator produced highly consistent results, yielding a pooled mean of 17.76 (95% CI 15.05–20.47) and similar heterogeneity (I^2^ = 90.19%). Leave-one-out analyses further supported the stability of the findings: when each data set was omitted in turn, the pooled mean varied only minimally between 16.93 and 18.42, indicating that the pooled estimate remained broadly stable across omissions.

**Fig. 6:**
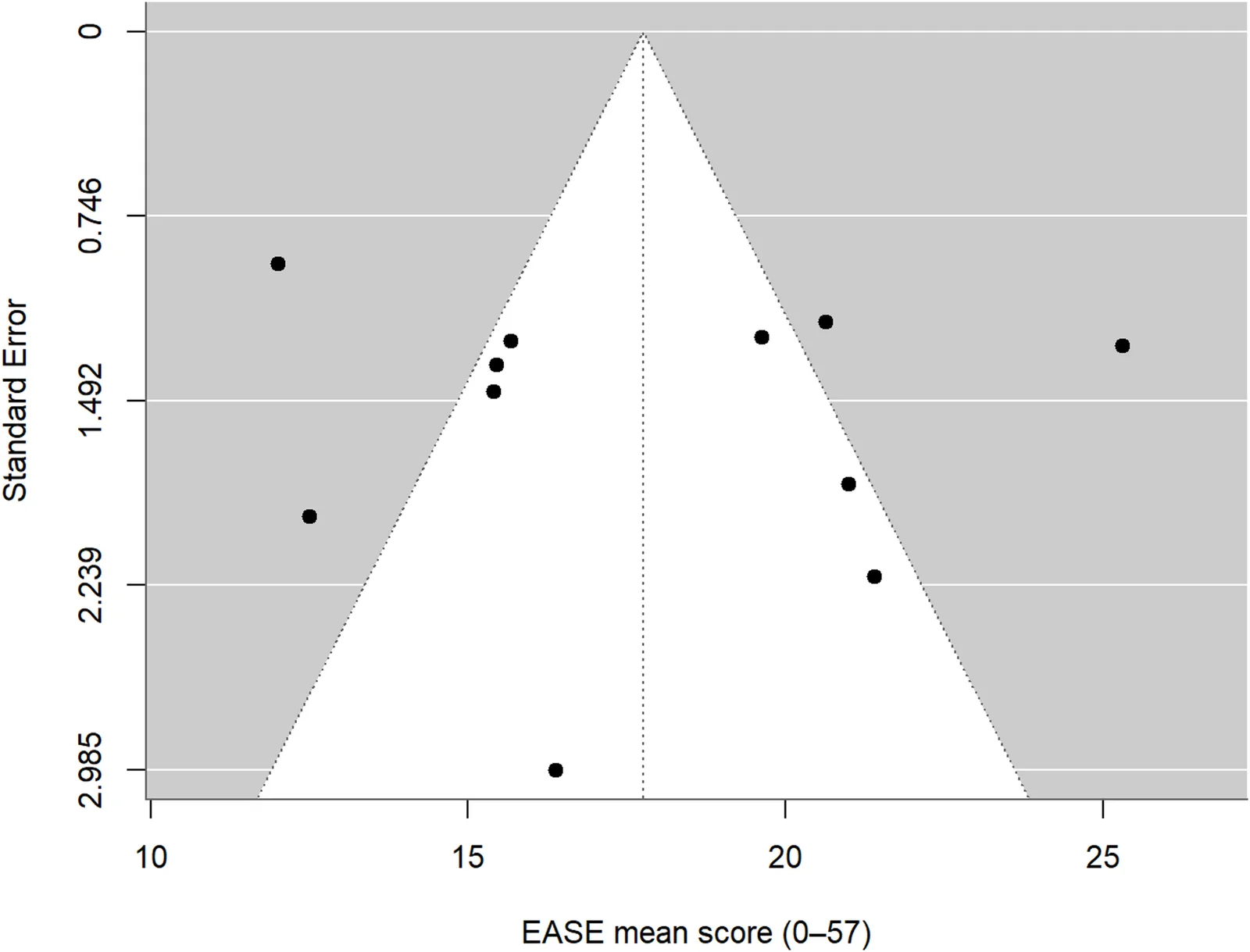
EASE-D funnel plot

Visual inspection of the funnel plot did not indicate marked asymmetry, although study estimates were widely dispersed around the pooled mean. The Egger-type regression test was non-significant (z = 0.096, *p* = 0.923), and the Begg-Mazumdar rank correlation test likewise indicated no evidence of small-study effects (τ = −0.018, *p* = 1.000). No data sets were imputed under the trim-and-fill procedure (k₀ = 0). The adjusted pooled mean remained unchanged at 17.76 (95% CI 15.29–20.24), strengthening confidence in the robustness of the overall estimate. A funnel plot for meta-analysed EASE-D studies is presented in Fig. 6.

Subgroup analyses by clinical setting suggested possible variation in mean EASE-D scores. Community-based samples showed the lowest pooled mean (14.96, 95% CI 13.30–16.62; *k* = 4), whereas inpatient samples reported higher levels of self-disturbance (20.32, 95% CI 18.76–21.87; *k* = 3). Mixed inpatient/community samples (20.48, 95% CI 11.05–29.92; *k* = 2) and studies with unspecified settings (16.36, 95% CI 7.55–25.18; *k* = 2) had notably wider CIs, potentially reflecting sampling uncertainty. The small number of studies within each subgroup substantially limits the precision and interpretability of these comparisons.

Examining differences between applied diagnostic labels showed higher mean scores in schizophrenia labelled samples (20.71, 95% CI 14.97–26.45; *k* = 3) compared to UHR samples (13.26, 95% CI 11.10–15.43; *k* = 3). However, the small number of studies cautions against strong interpretations. Country-level subgrouping revealed pooled means of 16.04 in Italy (95% CI 10.96–21.11; *k* = 3), 20.39 in Norway (95% CI 10.73–30.04; *k* = 2), and 20.32 in Denmark (95% CI 18.76–21.87; *k*= 3). As with the DES, the small number of contributing studies per country and methodological differences between samples restrict interpretive value. 

Exploratory univariable meta-regressions assessing continuous study-level predictors identified publication year as a significant moderator of mean EASE-D scores (β = −0.627, 95% CI −1.194 to -0.060, p = .030). Mean age (β = 0.210, 95% CI −0.322 to 0.741, p = .440) was unrelated to total EASE-D score. Gender distribution (β = −0.117, 95% CI −0.236 to 0.001, p = .052) and sample size (β = 0.146, 95% CI −0.018 to 0.311, p = .081) showed trend-level associations, although neither reached conventional statistical significance. Sample size (β = 0.146, 95% CI -0.018 to 0.311, p = .081) showed a trend-level association, explaining approximately 19% of between-study variance, whereas publication year explained approximately 31% of between-study variance. Overall, these analyses suggest that the examined methodological and demographic factors explain some between-study variability in mean EASE-D scores, although substantial heterogeneity remained unexplained. 

### Meta-analysis of the severity of anomalous self-experience in non-affective psychosis by EASE-C score

A total of five studies contributing six independent data sets were included in the meta-analysis of anomalous self-experience severity measured using the continuous scoring method of the EASE, comprising 235 participants with non-affective psychosis and related clinical risk states. Studies were conducted across three countries (Australia, the UK, and Italy) and were published between 2012 and 2021. Samples were drawn primarily from community settings. One study did not specify whether participants were recruited from inpatient or community services, with the service they recruited from containing both inpatient and community care pathways [187]. Details for the studies included in the EASE-C meta-analysis are presented in Table 5.

**Table 5:** Studies included in EASE-C meta-analysis

Study	Country	Setting	Diagnostic group	Sample size	EASE Continuous Mean and SD
Bonoldi et al. [188]	UK	Community	UHR	31	117.3 (SD = 68.6)
Comparelli et al. [189]	Italy	Community	UHR	45	51.09 (SD = 4.4)
Nelson et al. [187]	Australia	Does not specify	UHR	49	45.02 (SD = 26.23)
Nelson et al. [190]	Australia	Community	UHR / FEP	50 / 39	63.3 (SD = 34.65) / 78.74 (SD = 29.82)
Spark et al. [191]	Australia	Community	UHR and FEP schizophrenia spectrum	21	101.52 (SD = 30.43)

**Fig. 7:**
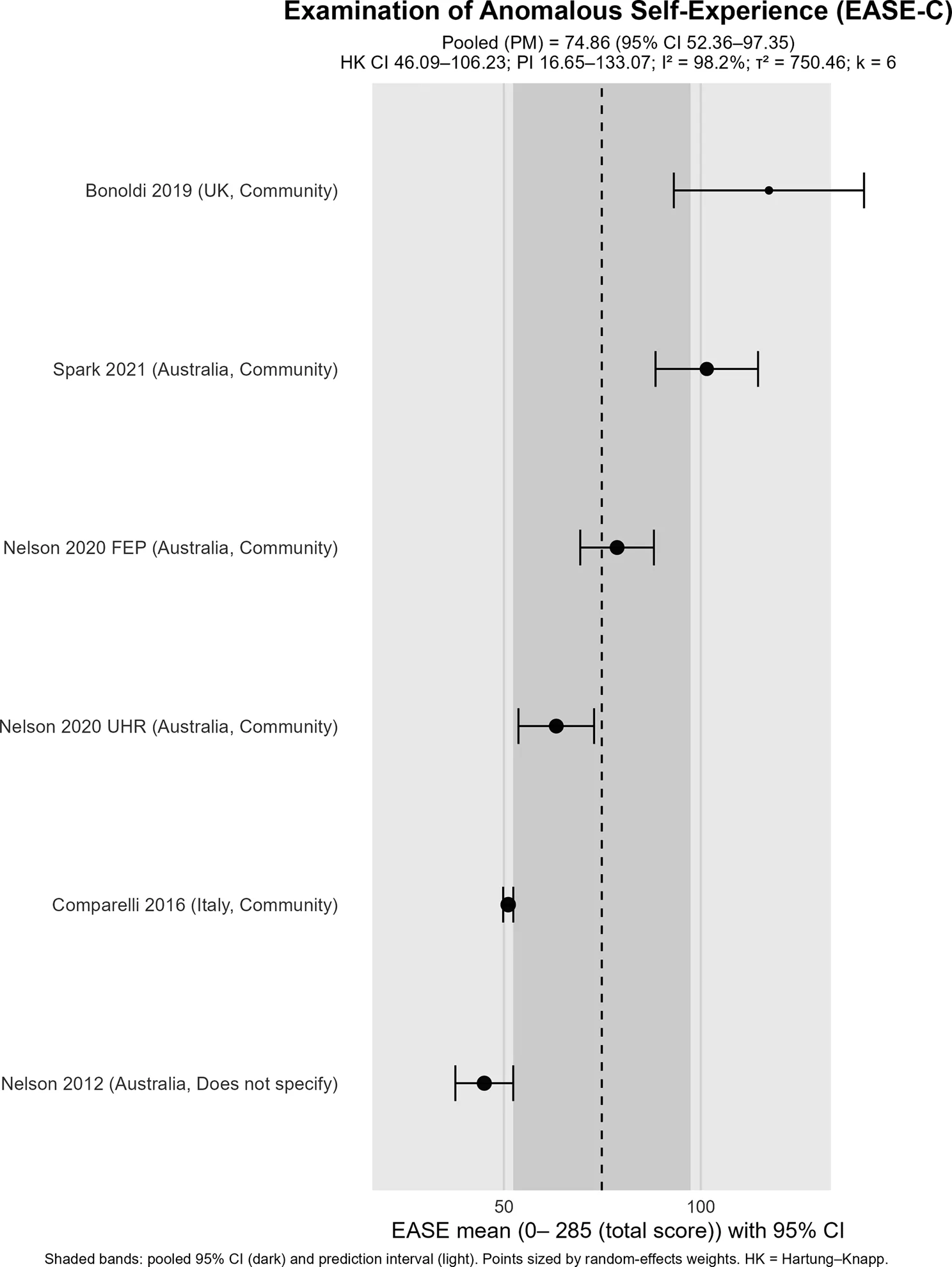
EASE-C forest plot

A random-effects model using the PM estimator yielded a pooled mean EASE-C score of 74.86 (95% CI 52.36–97.35). As with EASE-D scores, this indicates a substantial, clinically relevant load of self-disturbance experiences. A H-K adjustment produced a wider and more conservative CI (46.09–106.23), though the point estimate remained similar (76.24). Between-study heterogeneity was extremely high (I^2^ = 98.2%, τ^2^ = 750.2), indicating substantial variation in the load of anomalous self-experience. This very high heterogeneity indicates that estimates of EASE-C scores vary considerably and should be interpreted with caution, as they may plausibly be reflecting the small evidence base and methodological variation across studies. A forest plot for meta-analysed EASE-C studies is presented in Fig. 7.

Sensitivity analyses demonstrated excellent stability of the pooled estimate across variance estimators (PM: 74.86 95% CI 52.36–97.35; REML: 74.82 95% CI 52.66–96.90; DL: 73.63 95% CI 58.09–89.17). These nearly identical pooled values indicate that the results are not dependent on the choice of τ^2^ estimator.

Leave-one-out diagnostics showed that removing any single data set produced pooled means in a narrow range, with all resulting CIs overlapping substantially. The overall estimate varied by less than ±10 points, and no study produced a noticeable shift in the pooled interpretation. Influence diagnostics further indicated that no individual data set exerted disproportionate influence on the model. Although variability in residual measures reflected small-sample sensitivity, no single data set showed outlier behaviour sufficient to distort the pooled effect. Overall, this supports the robustness of the results despite high heterogeneity.

## Discussion

The present research aimed to establish the severity of dissociation and dissociative phenomena in non-affective psychosis and explore potential influences on severity. In doing so, it provides much-needed evidence on dissociative experiences as potential therapeutic targets that are closely linked to psychotic symptomatology and multiple adverse clinical outcomes.

### Summary of key findings

Meta-analysing the severity of broad dissociation in non-affective psychosis via DES scores indicated that dissociative phenomena are moderately elevated and highly heterogeneous amongst individuals with non-affective psychosis (*M* = 21.28, I^2^ = 97.29%). This suggests clinically relevant, albeit markedly varied broad dissociative experiences within this population.

Using CDS scores, meta-analysis of the severity of DP/DR in non-affective psychosis indicated moderately elevated detachment-style dissociative symptoms (*M* = 36.38 I^2^ = 91.13%) which exceed typical community levels but fall below levels suggestive of severe DP/DR or DP/DR disorder [36]. Nonetheless, wide H-K adjusted 95% CIs (23.59–51.98) indicate that severity differs substantially within non-affective psychosis populations, with some individuals reaching more clinically severe levels.

Meta-analysing the severity of anomalous self-experience in non-affective psychosis via the EASE instrument demonstrated a high and clinically significant load of self-disturbance across both scoring approaches. Using the dichotomous method, non-affective psychosis samples endorsed, on average, approximately 18 out of 57 anomalous self-experiences, suggesting a broad and pervasive disruption in sense of self. Pooled continuous scores reflected comparably elevated self-disturbance levels, equating to on average around 15 out of 57 anomalous self-experiences. These scores substantially exceed those reported in other diagnostic groups and healthy controls [186]. True mean severity also varied widely across samples (I^2^ = 88.2–98.2%), underscoring that, like broad dissociation and DP/DR, degree of self-disturbance differs markedly across non-affective psychosis samples.

In addition to meta-analysed findings, studies reported severity scores using a wide range of validated dissociation measures that did not meet the threshold for meta-analysis (*k* = 19). Despite high methodological variance, a consistent pattern emerged: non-affective psychosis samples, on average, score more highly on measures of dissociative experiences than healthy controls. In some cases, mean scores overlapped with or exceeded thresholds used to indicate clinical significance or diagnostic relevance in other populations. However, the interpretive value of these findings was often constrained by limited uptake of certain measures within psychosis populations and a subsequent lack of established severity thresholds or normative thresholds specific to non-affective psychosis.

Included studies demonstrated moderate methodological quality overall. Strong measurement strategies and broadly appropriate analytic approaches were utilised across studies, but limitations included unclear recruitment flow and a lack of confounder control. Reduced representativeness due to risk-associated exclusions of the most severely unwell participants is noted but nonetheless is in keeping with ethical and clinically justifiable practice.

### Comparisons to literature

The meta-analysed DES mean of 21.28 aligns with prior estimates, though notably places dissociation over the moderate threshold of 20 according to clinical DES scoring guidance [192]. One review reported a pooled DES mean of 19.66 in SSD [13], and another reported a slightly lower value of 17.8 in those diagnosed with schizophrenia [15]. Renard et al. [13] do not report heterogeneity or CIs, whereas Lyssenko et al. [15] found a slightly larger 95% CI (15.6–20.2), and slightly lower, though still high heterogeneity (I^2^ = 80.5). Differences might plausibly reflect the narrower inclusion criteria and smaller *k*’s of both studies, perhaps especially in Lyssenko et al. [15], which employed tighter diagnostic restrictions (Lyssenko *k* schizophrenia = 17; Renard *k* SSD = 30). A further influence might be the effects of additional evidence generated in the time elapsed.

Comparing the DES pooled mean of 21.28 with other diagnoses indicates elevated scores above affective disorders such as depression (*M* = 15.3) and anxiety (*M* = 15.2), but below prototypical dissociative disorders (*M* = 38.9), PTSD (*M* = 28.6), and borderline personality disorder (*M* = 27.9) [15]. The updated mean of the present review also moves the mean DES score of non-affective psychosis above those reported for feeding and eating disorders (*M* = 18.6), gambling (*M* = 19.9), and alcohol use disorder (*M* = 19.7) in Lyssenko et al.’s [15] review.

Meta-regressions indicated that publication year was the only study-level characteristic that remained independently associated with DES scores in the adjusted model, with more recently published studies tending to report higher levels of dissociation. However, the omnibus test for this model approached (p = 0.059), but did not reach, conventional statistical significance, meaning that the adjusted findings should be interpreted cautiously. Mean age was associated with DES scores in univariable analyses but did not remain significant after adjustment for other study characteristics, suggesting that this relationship was not independent.

The observed publication-year effect was modest and explained only a small proportion of the substantial heterogeneity observed across studies. It is indeterminable whether this reflects a genuine increase in dissociation severity over time, or whether this pattern reflects shifts in how samples are constructed and/or how dissociative experiences are conceptualised and measured. Nonetheless, it is notable that more recent studies have increasingly been conducted within trauma-informed clinical and theoretical frameworks [27, 42, 193–196], which may influence participant selection and the recognition of dissociative experiences. It is also possible that more recent samples differ in trauma exposure or in willingness to report dissociative phenomena [197]. 

Interestingly however, a comparable publication-year effect was observed within the EASE-D meta-analysis, although in the opposite direction, with more recently published studies tending to report lower levels of anomalous self-experience. Given the small number of studies contributing to these analyses, it remains unclear whether these temporal patterns across measures reflect genuine differences in clinical severity or broader changes in sampling, measurement, and service contexts over time. Differences in the direction of the publication-year effects observed in the DES and EASE-D analyses might also reflect a fundamental difference in the constructs being measured. Although self-disorder may be considered as a dissociative experience, particularly insofar as it aligns with depersonalisation, it is not synonymous with broad dissociation. The directional divergence may therefore plausibly reflect this distinction. These explanations remain speculative yet highlight that clinical severity estimates are shaped not only by psychological processes but also by changing recruitment strategies, measurement choices, and service contexts.

It is also noteworthy that diagnostic terminology is applied inconsistently across the included literature, with samples variously labelled as schizophrenia, SSD, or related groupings under the non-affective psychosis umbrella. Within the present review, diagnostic labels were either explicitly assigned within included articles, matched as closely as possible to reported demographics, or derived from the article’s inclusion criteria. The variation found likely reflects differences in researcher background, localised naming conventions, and recruitment practices. FEP, for example, is commonly used as a service category well beyond the first episode of psychosis a person experiences [198], and broader diagnostic categories such as SSD encompasses multiple psychotic diagnoses and subtypes, including schizophrenia itself. As a result, these labels often index overlapping clinical populations, undermining their capacity to delineate discrete subgroups for comparative analysis. Despite these limitations, examining dissociation across diagnostically defined subgroups remains informative, as such labels are routinely assumed to indicate meaningful differences in research and clinical practice [199–201].

In actuality, subgroup analyses indicated that diagnostic grouping showed little capacity to account for variability in dissociative symptom severity. If diagnostic labels did reflect discrete, stable clinical categories, some systematic variation in dissociation levels might be expected. Instead, dissociation levels were broadly comparable across the various labels applied, with substantial heterogeneity persisting within each categorisation. This convergence across differently named samples suggests that dissociative phenomena do not map variably across diagnostic naming conventions, aligning with longstanding concerns regarding the classification of psychoses more broadly [202, 203]. Notably, the only clear departure from this pattern emerged in comparisons involving samples categorised as UHR, where levels of anomalous self-experience were demonstrably lower than in schizophrenia labelled samples. UHR groups are defined by risk and developing symptoms, and often capture earlier, more variable presentations [204]. Plausibly, dissociative experiences may therefore be less persistent or severe in this prodromal stage [57, 205], meaning any deviation from the broader pattern would be most likely to emerge around this distinction.

The limited explanatory value of diagnostic labels for dissociation in non-affective psychosis appears to reflect a poor fit between categorical diagnostic systems and the phenomenology of dissociative experiences. Dissociation may therefore be better understood as a transdiagnostic process, more effectively measured and considered via dimensional, mechanism-focused means. However, this does not imply that diagnostic categories are redundant as organisational or clinical tools. Within current health systems, categorical frameworks retain value by enhancing reliability, supporting communication, and providing a shared reference point for clinical decision-making [206, 207]. Whilst a transition to momentary-assessment-based diagnoses of psychoses has been proposed by some [208, 209], the present findings are likely to be operationalised within existing diagnostic frameworks, conceptually constrained though they may be. As such, the challenge is not to discard existing diagnostic frameworks, but to work within them whilst applying dimensional and mechanism-focused approaches to dissociative phenomena and potentially to psychosis more broadly.

Turning specifically to depersonalisation and derealisation, studies using the CDS reported that, whilst elevated relative to community controls, DP/DR severity, on average, remained below the threshold typically used to identify clinical-level DP/DR [36]. Plausibly, this reflects the CDS’s narrow focus on detachment-type dissociative experiences and its calibration against populations with more severe DP/DR. Nonetheless, transdiagnostic research confirms that DP/DR is relatively common in both psychosis and other mental health conditions, suggesting that DP/DR should be routinely assessed even if it does not consistently reach apparent disorder-level threshold [13, 210].

 EASE-D meta-analysis results (M= 17.76 95% CI 15.29–20.24) align extremely closely with a recent transdiagnostic meta-analysis of basic self-disorder, which reported a total EASE-D mean score in schizophrenia-spectrum psychosis of 17.81 (95% CI 15.17–20.45) [186]. Lower scores were reported in clinical high-risk populations (M = 13.89), non-schizophrenia spectrum psychoses (M = 8.64), affective disorders (M = 5.7), obsessive-compulsive disorder (M = 5.33), and healthy controls (M = 0.84) [186]. These pooled estimates are further corroborated by meta-analytic evidence showing large group differences between SSDs and other psychiatric groups and healthy controls (Hedge’s g typically > 1.5) [205, 211]. 

The presently reported pooled EASE-D and EASE-C means, indicating endorsement of between a quarter and one-third of anomalous self-experience items, therefore complement this broader evidence and underscore that self-disturbance is a prominent symptomatic feature within those who experience non-affective psychosis. Whilst continuous scoring of EASE suggests a slightly lower quantitative load of self-disturbance (just over a quarter versus a third), this likely reflects its more nuanced design which captures not only the presence of anomalous self-experiences but also their intensity. As such, lower total scores are expected via this approach and do not necessarily indicate less clinical relevance. Instead, they provide a more graded representation of self disturbance.

In addition to differences between schizophrenia and UHR samples, subgroup analyses for EASE-D found indications of higher self-disturbance scores in inpatient than community samples. In both cases, these comparisons were based on small numbers of studies and should therefore be interpreted cautiously. Although such explanations are tentative, the pattern of higher self-disturbance in schizophrenia compared with UHR samples aligns with accounts that conceptualise basic self-disturbance as a trait-like marker of schizophrenia-spectrum vulnerability that is evident prior to, and remains prominent following the onset of psychosis [57, 205]. It also aligns to contemporary clinical practices whereby inpatient care is typically reserved for more symptomatically acute presentations [212].

A final, particularly striking feature of the findings is the diversity of dissociation measures in use. This was shown by a recent COnsensus-based Standards for the selection of health Measurement INstruments (COSMIN) review which evaluated 44 dissociation scales and found wide variability in content coverage, methodological quality, and psychometric robustness [213]. Only a small subset, including the DES, PDEQ, SDQ, and CDS, had strong evidence for reliability and validity across contexts, whereas many measures were developed in trauma-exposed or dissociative disorder samples and only minimally validated elsewhere. The present findings replicate this pattern. A small number of instruments dominate, alongside many used in only one or two psychosis studies which are typically not validated for use in psychosis populations. Although dissociation is multidimensional and different instruments can capture distinct facets (e.g., detachment, compartmentalisation, self-disturbance), the proliferation of measures also creates conceptual and methodological fragmentation. Variation in construct focus, scoring format, and state versus trait emphasis constrains direct comparison and undermines attempts to unify empirical findings within coherent theoretical models, particularly in non-affective psychosis. Greater coordination in measurement, whilst retaining sensitivity to dissociation’s complexity, would strengthen efforts to advance the field.

### Conceptual challenges

One unresolved question is whether dissociation in non-affective psychosis reflects the same underlying mechanisms as in dissociative disorders or a partially distinct construct with areas of overlap. Although this review confirms that dissociation is elevated in non-affective psychosis, few studies directly compare psychosis and dissociative disorder presentations [13]. As a result, it remains unclear whether dissociation in psychosis reflects shared mechanisms, a psychosis-specific pathway, or a *true* comorbidity in a subset of individuals. Progress in this area will require systematic cross-diagnostic comparisons using consistent measurement. For now, dissociation is clearly an important clinical feature of psychosis, but its relationship to dissociative disorder remains to be resolved.

Beyond the ontological status of psychosis-*type* dissociation, the inclusion of the EASE raises questions about where basic self-disturbance sits relative to positive symptoms. Several EASE items, such as *hyperreflectivity*, *inability to discriminate modalities of intentionality*, or *solipsistic grandiosity* [37], align closely to experiences that in standard diagnostic systems fall under positive symptoms, i.e., paranoia, disorganised thinking, and delusions of grandeur [214]. This invites the concern that the EASE may be relabelling psychotic phenomena and prompts consideration of whether self-disturbance is a distinct construct or part of the broader positive symptom domain.

However, phenomenologically developed accounts argue strongly that basic self-disturbances are not simply psychotic symptoms described differently, rather, they reflect instability in *ipseity*, the implicit “mineness” of experience, which forms a persistent background vulnerability across SSDs [37]. Empirical findings broadly support this distinction. Anomalies in self-experience often emerge before the first episode and persist beyond remission [215], indicating that they do not map directly to psychotic symptom trajectories. They also show only modest associations with current positive symptoms and demonstrate high stability over time, including in at-risk mental states and during remission [211, 216]. This pattern suggests that basic self-disturbance represents a trait-like vulnerability that can precede, coexist with, and persist beyond psychotic symptoms, rather than being reducible to them.

### Clinical implications

In light of present findings, services which support non-affective psychosis populations should consider implementing routine assessment of dissociative experiences. Practically, brief psychosis-validated screeners, such as the G-FSA [21], could be used to support initial efficient identification. Where indicated, more detailed instruments, such as the 35-item ČEFSA [21], DES [35], or clinician-rated measures [38], could then be used to inform formulation and care planning. Given increasingly robust evidence that dissociation forms part of a trauma-related pathway to psychosis [26, 45, 46], trauma-informed formulation should explicitly integrate experiences of childhood adversity, dissociative symptoms, and psychotic phenomena. This aligns with trauma-informed care guidance for both psychosis and dissociative presentations [195, 217]. Importantly, dissociative presentations need not preclude an individual from trauma-focused therapy. One study showed imagery-rescripting work with trauma-linked voices led to reductions in intrusions and voice distress, even amongst clients who dissociated during treatment, albeit they required additional sessions to achieve equivalent benefit [218]. Such findings are consistent with practice-based guidance [219] suggesting that dissociative symptoms may necessitate adaptations to pacing, repetition, and scaffolding within therapy.

Although fully evaluated psychosis-specific interventions targeting dissociation are currently underdeveloped, early, small-scale clinical findings are encouraging. One small case-series study of a dissociation-focused cognitive-behavioural intervention demonstrated reductions in both dissociation and auditory hallucinations in people with active psychosis, with symptomatic improvements maintained at six-month follow-up [172]. Evidence from dissociative disorder and DP/DR treatment also shows that interventions focused on grounding, affect regulation, identity integration, and the re-appraisal of anomalous experiences can reduce distress, PTSD symptoms, functional impairment, and suicidality [220–222]. These variables are also implicated in psychosis [18], supporting research which positions dissociation as a plausible transdiagnostic intervention target even where experiences do not meet diagnostic thresholds or are not explicitly trauma-linked [210, 219]. In particular, affect intolerance and perceived limitations in self-efficacy have been highlighted as key background factors maintaining certain dissociative experiences, indicating that interventions which enhance emotional literacy, tolerance of internal states, and confidence in coping, may be especially relevant in therapy for people experiencing psychosis who dissociate [219].

Enhanced workforce development is nonetheless needed to support safe and effective implementation. To translate dissociation centred interventions into routine practice, clinicians require clear guidance and training, as confidence in recognising and managing dissociative experiences in psychosis remains limited [217]. Clinicians often possess the core CBT skills required to address dissociation, but typically lack explicit training or guidance in recognising dissociation as a distinct and clinically meaningful intervention target [219]. Embedding trauma-informed approaches at the service level would greater support safe implementation and ensure that dissociation is systematically addressed within care [195, 223]. 

### Limitations

Whilst three measures met the threshold for meta-analysis, the number of studies contributing to three out of four analyses was low to modest (CDS *k* = 6; EASE-C *k* = 6; EASE-D *k* = 11). This has the potential to constrain the precision of pooled estimates and precludes more granular subgroup or multivariable meta-regression for some of these instruments [68, 69]. Subgroup estimates for DES and EASE-D were sometimes based on relatively small numbers of studies in some categories and should therefore be treated as exploratory. Within the meta-analysis of DES scores, a small number of included studies reported symptomatically varied subgroups, such as hallucinating versus non-hallucinating participants, or those using substances versus those not using substances. These subgroups presented with variability in dissociation severity, as such, higher-scoring subgroups may have influenced that study’s mean. Nonetheless, because the aim was to estimate overall dissociation within non-affective psychosis rather than symptom-state or environmental differences, pooling these subgroups remained a methodologically valid approach.

In addition, although the inclusion criteria focused on non-affective psychosis, a small number of studies recruited diagnostically mixed samples and were retained by applying a ≥ 50% non-affective psychosis threshold (*k* = 10). This approach optimised yield but also introduced some small diagnostic heterogeneity. More widely, although the search strategy was extremely inclusive, spanned six major databases, and was supplemented by citation searching, no review can guarantee complete retrieval. Four full texts were irretrievable and, despite attempts to contact authors, 21 studies meeting inclusion criteria could not be included in meta-analyses due to unextractable data or data which was not suitable for the conducted meta-analyses. Inclusion was also restricted to peer-reviewed publications using validated measures, excluding potentially relevant grey literature or preliminary data. Nonetheless, this approach ensured robust quality control of evidence, which is essential for clinically focused reviews.

A final consideration concerns how severity of dissociation was operationalised. Consistent with standard meta-analytic practice [49], severity was indexed using study-level mean total scores on validated dissociation measures, which provide established indicators of symptom load. Nonetheless, dissociation scales differ in the extent to which they capture frequency, intensity, impact, and breadth of phenomena [213]. Severity estimates in the present review should therefore be interpreted as comparative indicators of symptom load within each measure. Differences in construct focus and scaling mean that scores are best understood within, rather than compared across instruments.

## Conclusions

The present systematic review and meta-analyses show that the severity of dissociation and dissociative phenomena is, on average, moderately to markedly elevated in non-affective psychosis. This represents the first comprehensive quantification of dissociative severity across multiple instruments in non-affective psychosis, highlighting the substantial variation in measurement approaches throughout the literature and the associated conceptual complications. Framed within wider research, the data highlight the clinical relevance of dissociative phenomena in non-affective psychosis and their potential importance within broader models linking trauma, dissociation, and psychotic symptoms. They draw attention to the pressing need for more harmonised measurement efforts, mechanistic research, and the development of trauma-informed, dissociation-targeted clinical interventions. Particularly important questions for future research include how dissociative phenomena interact with psychotic symptoms over time, whether they contribute directly to symptom persistence, distress, functional outcomes, and suicide-related outcomes, as well as the extent to which these relationships vary across stages of illness and clinical presentations.

## Electronic supplementary material

Below is the link to the electronic supplementary material.

Supplementary Material 1: S1. Inter-rater reliability at title and abstract.csv

Supplementary Material 2: S2. Inter-rater reliability at full text.csv

Supplementary Material 3: S3. Quality assessment of included studies (studies included in quantitative analysis only).csv

Supplementary Material 4: S4. Quality assessment of included studies (studies included under wider review criteria).csv

## Data Availability

Meta-analytic data are available from the corresponding author upon reasonable request.

## Abbreviations

BLIPS: Brief Limited Intermittent Psychotic Symptoms
CDS: Cambridge Depersonalization Scale
CI: Confidence Interval
CTQ: Childhood Trauma Questionnaire
DDD: Depersonalisation/Derealisation Disorder
DES: Dissociative Experiences Scale
DL: DerSimonian-Laird
DP/DR: Depersonalisation/Derealisation
DSM-V: Diagnostic and Statistical Manual of Mental Disorders, Fifth Edition
EASE: Examination of Anomalous Self-Experience
EASE-C: Examination of Anomalous Self-Experience (Continuous Scoring)
EASE-D: Examination of Anomalous Self-Experience (Dichotomous Scoring)
FEP: First-Episode Psychosis
FSA: Felt Sense of Anomaly
H-K: Hartung-Knapp
ICD: International Classification of Diseases
MMAT: Mixed Methods Appraisal Tool
PANSS: Positive and Negative Syndrome Scale
PI: Prediction Interval
PM: Paule-Mandel
PRISMA: Preferred Reporting Items for Systematic Reviews and Meta-Analyses
PROSPERO: International Prospective Register of Systematic Reviews
PTSD: Post-Traumatic Stress Disorder
RCT: Randomised Controlled Trial
REML: Restricted Maximum Likelihood
SD: Standard Deviation
SSD: Schizophrenia Spectrum Disorder
UHR: Ultra-High Risk
